# Therapeutic strategies for MMAE‐resistant bladder cancer through DPP4 inhibition

**DOI:** 10.1002/1878-0261.70187

**Published:** 2025-12-21

**Authors:** Gang Li, Shuichi Tatarano, Hirofumi Yoshino, Saeki Saito, Mitsuhiko Tominaga, Junya Arima, Ikumi Fukuda, Takashi Sakaguchi, Ryosuke Matsushita, Yasutoshi Yamada, Hideki Enokida

**Affiliations:** ^1^ Department of Urology, Graduate School of Medical and Dental Sciences Kagoshima University Japan

**Keywords:** AKT, bladder cancer, dipeptidyl peptidase 4, monomethyl auristatin E, sitagliptin

## Abstract

Monomethyl auristatin E (MMAE) is used as the cytotoxic payload for enfortumab vedotin (EV) in the treatment of locally advanced and metastatic bladder cancer (BC). However, the development of resistance to MMAE in BC is a therapeutic problem. To explore the mechanism of resistance to MMAE in BC, we established MMAE‐resistant BC cells (MR‐BCs). RNA sequencing analysis showed that the expression of dipeptidyl peptidase 4 (DPP4, also called CD26) increased significantly in MR‐BCs compared with parental BC cells. Knock down of *DPP4* expression using small interfering RNA inhibited the viability of MR‐BCs. In addition, the DPP4 inhibitor sitagliptin suppressed the proliferation, migration, and invasion of BC cells, and cotreatment with MMAE effectively induced cell apoptosis, arrested cells in the G_2_M phase of the cell cycle, increased reactive oxygen species production by inhibiting the AKT pathway, and significantly inhibited the *in vivo* growth of MMAE‐resistant cells. This study provides insights into the use of DPP4 inhibitors as a treatment strategy for MMAE‐resistant BC.

## Graphical abstract

We established monomethyl auristatin E (MMAE)‐resistant bladder cancer (BC) cell lines by exposure to progressively increasing concentrations of MMAE *in vitro*. RNA sequencing showed *DPP4* expression was increased in MMAE‐resistant BC cells. Both si‐*DPP4* and the DPP4 inhibitor sitagliptin suppressed the viability of MMAE‐resistant BC cells. Sitagliptin also reversed the resistance to MMAE by restoring inhibition of the AKT pathway.

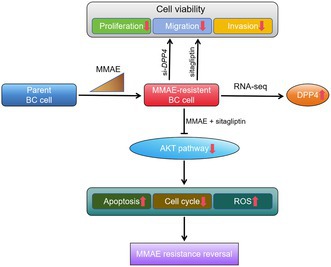

**Abbreviations**
ADC: antibody‐drug conjugate
BC: bladder cancer
DPP4: dipeptidyl peptidase 4
EV: enfortumab vedotin
FPKM: fragments per kilobase of exon per million fragments mapped
GO: Gene Ontology
IC_50_
: half‐maximal inhibitory concentration
KEGG: Kyoto Encyclopedia of Genes and Genomes
MMAE: monomethyl auristatin E
OS: overall survival
qRT‐PCR: quantitative reverse transcription polymerase chain reaction
ROS: reactive oxygen species
siRNA: small interfering RNA
TCGA: The Cancer Genome Atlas

## 1 Introduction

According to global cancer statistics reported in 2020, bladder cancer (BC) is the 10th most commonly diagnosed cancer worldwide [1]. It is predicted that by 2040, the global cases of BC and BC‐related deaths will increase by 73% and 87%, respectively, compared with those in 2020 [2]. To date, the combination of gemcitabine and cisplatin (GC) [3] and the combination of methotrexate, vinblastine, doxorubicin, and cisplatin [4] has been the standard BC chemotherapy regimens. Recently, multiple new chemotherapy drugs have become available clinically for the treatment of BC, including immune checkpoint inhibitors targeting the programmed death 1/programmed death ligand 1 axis, such as pembrolizumab [5], avelumab [6], and atezolizumab [7]; fibroblast growth factor receptor inhibitors, such as erdafitinib [8]; and antibody‐drug conjugates (ADCs), such as enfortumab vedotin (EV) [9] and sacituzumab govitecan [10]. A recent study found that EV and pembrolizumab (EV‐P) combination therapy significantly improves outcomes for patients with BC compared with traditional chemotherapy [11]. In addition, chemotherapy drugs have been used as adjuvant [12] and neoadjuvant [13] therapy for surgery and have been shown to effectively improve the prognosis of bladder cancer patients.

Monomethyl auristatin E (MMAE), the main component of EV, is one type of mitotic inhibitor that shares a mechanism of action with traditional taxane chemotherapeutics [14]. MMAE interferes with the formation of microtubules by binding to tubulin dimers, thereby inhibiting cell mitosis, leading to G_2_/M phase arrest, and inducing apoptosis [14, 15]. EV is an ADC‐targeting nectin‐4 that was approved in December 2019 by the United States Food and Drug Administration. MMAE is used as a cytotoxic payload for EV in the treatment of locally advanced and metastatic BC [16]. One study showed that compared with standard chemotherapy, patients treated with EV showed a reduced risk of death by 30% and significantly reduced median overall survival (OS) (12.91 versus 8.94 months) [17]. In addition, MMAE is also used as a treatment for multiple types of carcinoma, including lymphoma [18] and breast cancer [19], and has achieved good therapeutic effects.

In our previous studies, we found that the expression of *EHHADH* was increased in cisplatin‐resistant BC cells and that knockdown of *EHHADH* effectively inhibited the viability of parental and cisplatin‐resistant BC cells [20]. Similarly, the expression of *SMARCD1* was increased in gemcitabine‐resistant BC cells, and after knockdown of *SMARCD1*, the viability of parental and gemcitabine‐resistant BC cells was also significantly reduced [21]. In addition, we found that gemcitabine‐resistant and cisplatin‐resistant BCs did not exhibit cross‐resistance and that these BCs showed different mRNA patterns. Then, by inhibiting RAS‐dependent signaling, we also found that the pan‐RAS inhibitor Compound 3144 effectively inhibited the activity of gemcitabine‐ and cisplatin‐resistant BC cells [22]. However, few studies have examined MMAE‐resistant BC cells (MR‐BCs).

Since it is difficult to obtain clinical samples treated with ADCs with MMAE as payload, we established MR‐BCs *in vitro* and performed RNA sequencing analysis of parental BC cells and MR‐BCs. Based on the results, we identified target genes and performed related *in vitro* and *in vivo* analyses.

## 2 Material and methods

### 2.1 Cell lines, cell culture, and establishment of MR‐BCs *in vitro*

The cell lines T24 (RRID: CVCL_0554) and J82 (RRID: CVCL_0359) were purchased from the American Type Culture Collection (ATCC) (Manassas, VA, USA). These cell lines were authenticated as the same as the cell registered in ATCC by the Japanese Collection of Research Bioresources (JCRB) Cell Bank in 2023. All cell lines were tested and found negative for mycoplasma (e‐Myco Mycoplasma PCR Detection Kit; iNtRON Biotechnology, Seongnam, Korea). These cell lines were cultured in minimum essential medium (MEM) containing 10% fetal bovine serum (FBS), 100 units·mL^−1^ penicillin, and 100 μg·mL^−1^ streptomycin in a humidified environment consisting of 95% air and 5% CO_2_ at 37 °C. To establish MR‐BCs, MMAE (CAS NO. 474645‐27‐7; MyBioSource, San Diego, CA, USA) was added to the cells at increasing concentrations (4, 8, 16, 32, 64, 80, 100, and 128 nm) after cells had reached 30–50% confluence. After exposure to MMAE for 24 h, the MEM was replaced with fresh MMAE‐free MEM until the surviving cells recovered favorably. When cells reached the same confluence, MMAE was added to the MEM again. Cells were exposed to each concentration 6–8 times. After approximately 10 months, cells that grew in the MEM with 128 nm MMAE were designated MR‐T24 and MR‐J82 cells. These two MR‐BCs were stored for further analyses.

### 2.2 Determination of the IC_50_

The half‐maximal inhibitory concentration (IC_50_) was evaluated using XTT assays. Briefly, cells were seeded in 96‐well plates at 1000 cells per well in 90 μL MEM with 10% FBS and treated with 10 μL serially diluted concentration of MMAE or sitagliptin (CAS NO. 654671‐77‐9; MedChemExpress, Monmouth Junction, NJ, USA). After 96 h of incubation, cell proliferation was measured using a Cell Proliferation Kit II (Roche Diagnostics GmbH, Mannheim, Germany).

### 2.3 Cell proliferation, migration, and invasion assays

For cell proliferation assays, the cells were seeded in 96‐well plates at 1000 (treated with MMAE and/or sitagliptin) or 2000 (treated with si‐*DPP4*) cells per well. After 96 h of incubation, cell proliferation was measured using XTT assays, as described above.

Wound healing assays were performed to measure cell migration activity. T24/MR‐T24 cells at 2.0 × 10^5^ cells per well and J82/MR‐J82 cells at 3.0 × 10^5^ cells per well were seeded in 6‐well plates. After 48 h, a scratch was made in the cell monolayer using a P‐1000 micropipette tip. The initial gap length at 0 h and the residual gap length after 9 or 22 h were calculated from micrographs. Three microscopic fields were used for quantification.

For measurement of cell invasion activity, transwell invasion assays were performed using Matrigel invasion chambers (Corning Biocort, Bedford, MA, USA). Briefly, T24/MR‐T24 cells at 0.5 × 10^5^ cells per well and J82/MR‐J82 cells at 0.75 × 10^5^ cells per well were cultured on the cell culture inserts. After 48 h, cells that passed through the 8.0‐μm pores and adhered to the surface of the chamber were counted from micrographs. Eight randomized microscopic fields were used for quantification.

### 2.4 Apoptosis and cell cycle assays

Flow cytometric analysis of apoptosis and cell cycle was carried out using a cytoflex analyzer (Beckman Coulter, Brea, CA, USA) and cytexpert 2.4 software (Beckman Coulter). Compensation controls were prepared using single‐color stained samples, and auto‐compensation was applied using the cytoflex software.

For the apoptosis assay, cells were stained with fluorescein isothiocyanate (FITC) Annexin V and propidium iodide (PI) using an Apoptosis Detection Kit (BD Biosciences, San Jose, CA, USA). Cells were classified into four categories: viable cells, necrotic cells, early apoptotic cells, and late apoptotic cells, with 5000 events recorded per sample. Each experiment was repeated three times.

For the cell cycle assay, cells were stained with PI using a Cycletest Plus DNA Reagent Kit (BD Biosciences). DNA content was determined by taking the integrated intensity of each cell's fluorescent signal. Cells were divided into G0/G1 phase (first peak), S phase (between the G0/G1 and G2/M peaks), and G2/M phase (second peak), with 3000 events recorded per sample. Although the software output labels the fluorescence channel as ‘PE‐A’, this corresponds to the PI signal detected in the same channel configuration. Each experiment was repeated three times.

### 2.5 RNA sequencing analysis

RNA sequencing analysis was performed referring to the MINSEQE (Minimum Information about a high‐throughput SEQuencing Experiment) guidelines [23]. The parental BC cell lines were used as the reference material for the expression analysis. Total RNA from T24, J82, MR‐T24, and MR‐J82 cells was subjected to RNA sequencing, which was performed by Riken Genesis Co. Ltd (Kawasaki, Japan). RNA sequencing libraries were prepared using poly‐A selection and sequenced on the Illumina NovaSeq 6000 platform (2 × 101 bp, 50 million reads per sample). The reads were aligned to the GRCh37 reference genome with TopHat 2.0.13, and quantification was performed with cufflinks 2.2.1. The expression level was represented by fragments per kilobase of exon per million fragments mapped (FPKM). For gene expression comparison, only the genes test status with ‘OK’ (successfully tested in the RNA‐seq) were considered, while those marked as ‘FAIL’ (test failure) or ‘NOTEST’ (cannot be tested due to low alignment) were excluded.

### 2.6 *In silico* analysis

The Gene Ontology (GO) and Kyoto Encyclopedia of Genes and Genomes (KEGG) databases were analyzed using GeneCodis 4 (https://genecodis.genyo.es/). The Kaplan–Meier method was used to analyze OS by searching The Cancer Genome Atlas (TCGA) data through the OncoLnc dataset (http://www.oncolnc.org/). Comparisons of *DPP4* expression in BC samples of different pathological grades and clinical stages were analyzed by searching TCGA data through ucsc xena (https://xena.ucsc.edu/).

### 2.7 RNA extraction and qRT‐PCR

For total RNA extraction from the cells, ISOGEN (Nippon Gene, Tokyo, Japan) was used to prepare lysates of cultured cells according to the manufacturer's instructions. The quantitative reverse transcription polymerase chain reaction (qRT‐PCR) was performed as described previously [20, 21], with reference to the MIQE (Minimum Information for Publication of Quantitative Real‐Time PCR Experiments) guidelines [24]. The reference materials were the parental BC cell lines or the MMAE‐resistant BC cell lines. Beta glucuronidase (*GUSB*) or glyceraldehyde‐3‐phosphate dehydrogenase (*GAPDH*) were used as reference genes. Reactions (10 μL) were run for 45 cycles on a LightCycler 96 System (Roche) with no template controls. Relative gene expression was determined using the ΔΔ*C*_t_ method. Each experiment was repeated four times. The following primer sets were used to measure the mRNA expression levels by qRT‐PCR: *DPP4*, forward primer 5′‐GGGTCACATGGTCACCAGTG‐3′ and reverse primer 5′‐TCTGTGTCGTTAAATTGGGCATA‐3′; beta glucuronidase, forward primer 5′‐CGTCCCACCTAGAATCTGCT‐3′ and reverse primer 5′‐TTGCTCACAAAGGTCACAGG‐3′; and glyceraldehyde‐3‐phosphate dehydrogenase, forward primer 5′‐GGAGCGAGATCCCTCCAAAT‐3′ and reverse primer 5′‐GGCTGTTGTCATACTTCTCATGG‐3′.

### 2.8 Western blotting

Protein lysates were separated on NuPAGE gels using LDS Sample Buffer (Invitrogen, Thermo Fisher Scientific, Carlsbad, CA, USA). The western blot transfer system was used as described previously [20, 21]. Protein (10–30 μg per lane) was loaded onto gels. β‐actin was used as a housekeeping protein for normalization. Membranes were exposed for 5 s to 20 min. Each experiment was repeated three times. The following antibodies were used for immunoblotting: anti‐DPP4 (1 : 200; cat. no. ab215711; Abcam, Cambridge, UK), anti‐AKT (1 : 400; cat. no. 4691; Cell Signaling Technology, Danvers, MA, USA), anti‐phospho‐AKT (Ser473) (1 : 1000; cat. no. 4060; Cell Signaling Technology), anti‐Bcl‐xL (1 : 1000; cat. no. 2764; Cell Signaling Technology), and anti‐β‐actin (1 : 4000; cat. no. bs‐0061R; Bioss, Woburn, MA, USA). The secondary antibody was peroxidase‐conjugated anti‐rabbit IgG (1 : 5000; cat. no. 7074S; Cell Signaling Technology). Protein levels were assessed using imagej 1.48 (National Institutes of Health, Bethesda, MD, USA).

### 2.9 siRNA transfection

Cells were transfected with 50 nm small interfering RNA (siRNA) using Lipofectamine RNAiMAX transfection reagent (Thermo Fisher Scientific, Inc., Waltham, MA, USA) and Opti‐MEM (Thermo Fisher Scientific, Inc.). Transfection efficiency was evaluated by qRT‐PCR and western blotting with at least three replicates. A negative control siRNA was used as the negative control. The following siRNAs were performed: si‐*DPP4*‐1, sense 5′‐GGUUACCUUUGUUCCCAAAtt‐3′ and antisense 5′‐UUUGGGAACAAAGGUAACCtt‐3′ (cat. no. 104273; Ambion, Carlsbad, CA, USA); si‐*DPP4*‐2, sense 5′‐GGAUAAGAGGGAUUAGGGAtt‐3′ and antisense 5′‐UCCCUAAUCCCUCUUAUCCtg‐3′ (cat. no. 104274; Ambion); and negative control siRNA (cat. no. D‐001810‐10; Dharmacon, Horizon Discovery Group, Cambridge, UK). At 48 h after transfection, the cells were harvested and used in subsequent experiments.

### 2.10 ROS assays

Reactive oxygen species (ROS) levels were detected using a DCFDA/H2DCFDA Cellular ROS Assay kit (cat. no. ab113851; Abcam). Cells were seeded in 24‐well plates (1.0 × 10^5^ cells per well). After incubation at 37 °C and treatment with MMAE and/or sitagliptin for 24 h, the cells were washed with 1× buffer and then incubated with the diluted DCFDA solution at 37 °C for 45 min. Next, the cells were washed with 1× buffer again. The vehicle control group in each experiment served as the internal control. ImageJ software was used to quantify fluorescence intensity and calculate the integrated density of each image. Each experiment was repeated three times.

### 2.11 Xenograft model

All experiments described in this study were performed in accordance with the Regulations on Animal Experiments of Kagoshima University and were approved by the Kagoshima University Animal Experiment Committee (MD24071). Female nude mice (BALB/c nu/nu, 5 weeks old) were purchased from Charles River Laboratories (Yokohama, Japan). Mice were housed in rectangular cages (169 × 376 × 145 mm) under standard experimental conditions, including a 12‐h light/dark cycle at 23 ± 1 °C. The cages were lined with sawdust to provide water absorption and flexibility and were cleaned once a week. Mice had free access to water and a standard diet (Oriental Yeast CRF‐1, Tokyo, Japan). After the mice were acclimated, 1.0 × 10^7^ MR‐T24 cells were mixed with Matrigel (Corning) and injected subcutaneously into the flanks of the mice. After 2 weeks, the mice were divided into the phosphate‐buffered saline (PBS) group (*n* = 6), the MMAE (0.1 mg·kg^−1^) group (*n* = 6), the sitagliptin (200 mg·kg^−1^) group (*n* = 6), and the combination (MMAE 0.1 mg·kg^−1^ + sitagliptin 200 mg·kg^−1^) group (*n* = 6) and treated twice a week by intraperitoneal injection. Tumor size was calculated as 1/2 × length × width^2^. Weight and tumor measurements were performed twice a week, and mice were sacrificed on day 33.

### 2.12 Statistical analysis

Experiments were repeated at least three times, and the data were presented as means with error bars indicating standard deviations. Comparisons between two groups were analyzed using unpaired *t*‐test (parametric test) or Mann–Whitney *U*‐test (nonparametric test) after detecting normal distribution. For unpaired *t*‐test, Welch's *t*‐test was used when the variances were unequal. Comparisons between three or more groups were determined using one‐way analysis of variance (ANOVA) after detecting normal distribution, and Brown Forsythe and Welch ANOVA tests were used when the variances were unequal. Log‐rank tests were used for Kaplan–Meier survival curves. All analyses were conducted using graphpad prism 10.4.1 Software (GraphPad Software, Inc., San Diego, CA, USA).

## 3 Results

### 3.1 Establishment of MR‐BCs *in vitro* and analysis of the effects of MMAE on MR‐BCs

**Fig. 1:**
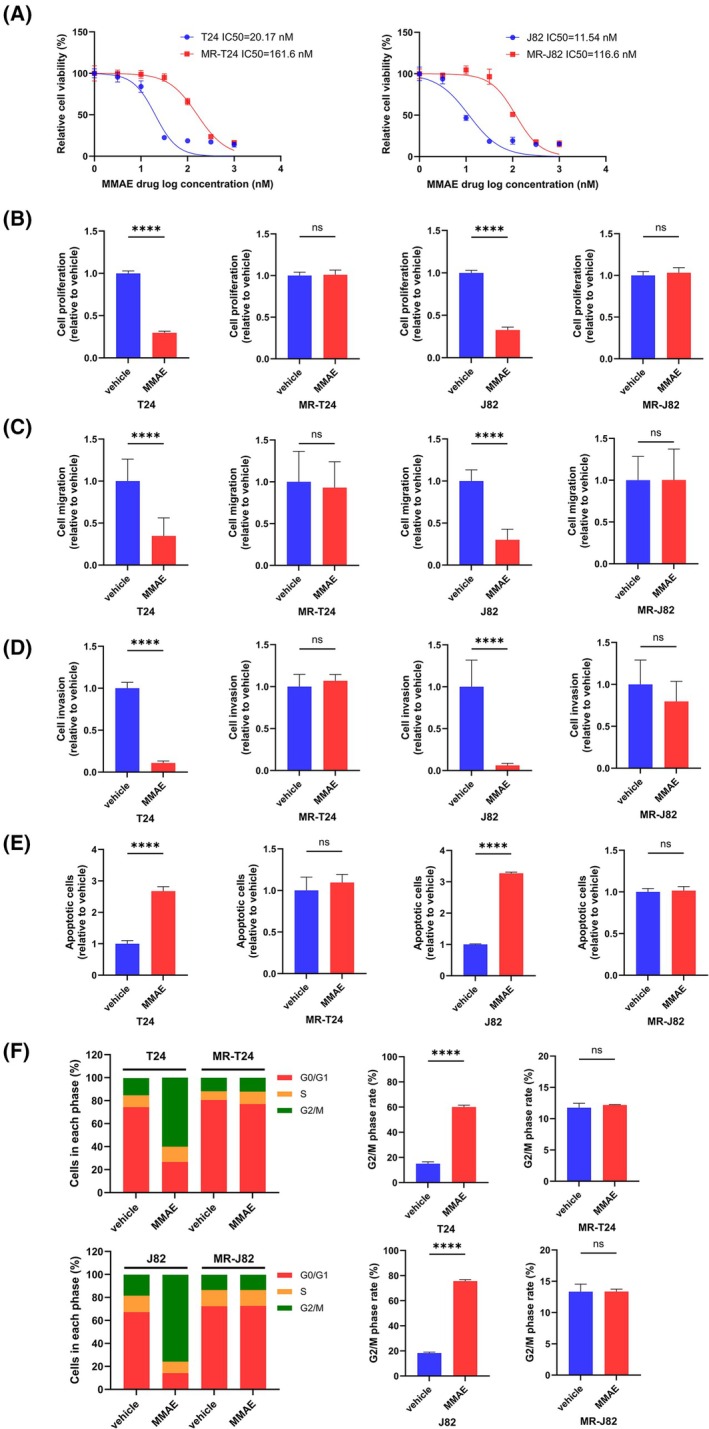
Establishment of MR‐BCs and effects of MMAE on BC cells. (A) IC_50_ values for T24/MR‐T24 cells and J82/MR‐J82 cells treated with MMAE (*n* = 4). (B) Cell proliferation according to XTT assay after MMAE treatment (*n* = 8). (C) Cell migration according to wound healing assay after MMAE treatment (*n* = 3). (D) Cell invasion according to Matrigel invasion assay after MMAE treatment (*n* = 8). (E) Apoptosis assay using flow cytometry after MMAE treatment (*n* = 3). (F) Cell cycle assay using flow cytometry after MMAE treatment (*n* = 3). MMAE concentration: 20 nm (T24/MR‐T24); 12 nm (J82/MR‐J82) (B–F). The statistical tests used were unpaired *t*‐tests (B–F). ns, no significance; ****P < 0.0001. The error bars indicate standard deviation (SD). MMAE, monomethyl auristatin E; MR‐J82, MMAE‐resistant J82; MR‐T24, MMAE‐resistant T24.

We established MMAE‐resistant T24 (MR‐T24) and MMAE‐resistant J82 (MR‐J82) cells. The IC_50_ was 8‐fold higher in MR‐T24 cells (161.6 nm) than in T24 cells (20.17 nm) and 10‐fold higher in MR‐J82 cells (116.6 nm) than in J82 cells (11.54 nm) (Fig. 1A). Then, we compared the effects of MMAE on parental BC cells and MR‐BCs. Cell proliferation assays were evaluated and we found that MR‐BCs had overcome the impact of MMAE (Fig. 1B). Cell migration and cell invasion assays also confirmed that the effects of MMAE on resistant cells had disappeared (Fig. 1C,D and Figs S1 and S2). Cell apoptosis assays showed that MMAE significantly increased apoptosis in parental BC cells, whereas apoptosis in MR‐BCs did not change (Fig. 1E and Fig. S3). In addition, cell cycle assays indicated that MMAE arrested parental BC cells in the G_2_/M phase, whereas MR‐BCs overcame this inhibitory effect (Fig. 1F and Fig. S4).

### 3.2 *DPP4* expression was significantly increased in MR‐BCs

**Fig. 2:**
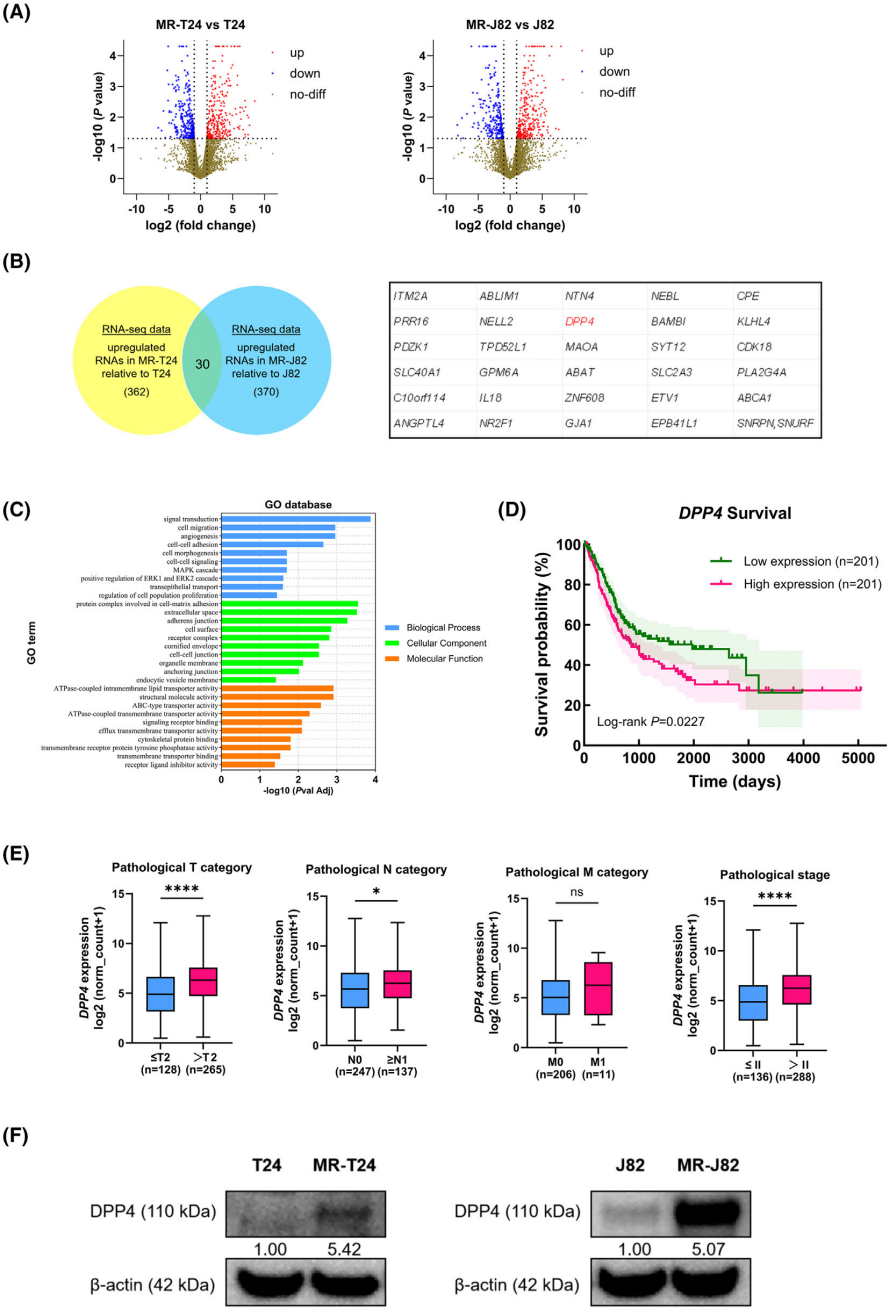
The relationship between *DPP4* expression and MMAE resistance in BC. (A) Volcano plot indicating gene expression differences between parental cells and MR‐BCs. (B) Venn diagram indicating the numbers of genes upregulated in MR‐BCs compared with parental cells (left) and the 30 genes significantly upregulated in both drug‐resistant cell lines (right). (C) GO analysis of 702 upregulated genes in MR‐BCs. (D) Kaplan–Meier survival curves for patients with BC exhibiting high (*n* = 201) or low (*n* = 201) *DPP4* expression. (E) Relationships between *DPP4* expression and pathological stages in samples from patients with BC. (F) DPP4 protein levels in parental BC cells and MR‐BCs, as determined by western blotting (*n* = 3). The statistical tests used were a log‐rank test (D) and a Mann–Whitney *U* test (E). ns, no significance; *P < 0.05; ****P < 0.0001. The error bars indicate SD. GO, Gene Ontology; MR‐J82, MMAE‐resistant J82; MR‐T24, MMAE‐resistant T24.

We performed RNA sequencing analysis to compare gene expression levels between MR‐BCs and parental BC cells. Then, we screened genes with significantly increased expression in MR‐BCs compared with parental cells using the following screening criteria: (a) test status ‘OK’, (b) *P* value less than 0.05, (c) log2(fold_change) greater than 1 (Fig. 2A, Table S1). Venn diagram analysis showed that 702 genes were significantly upregulated in MR‐BCs (Fig. 2B). Among these genes, 362 were upregulated in MR‐T24 cells compared with T24 cells and 370 were upregulated in MR‐J82 cells compared with J82 cells. Moreover, 30 genes were significantly increased in both types of MR‐BCs. GO analysis revealed that the 702 upregulated genes were associated with cell signal, cell surface, cell migration, and cell adhesion (Fig. 2C). KEGG analysis showed that the genes were related to the phosphatidyl inositol 3‐kinase (PI3K)/AKT pathway and cholesterol metabolism (Fig. S5). Among the 30 genes that were both significantly upregulated in MR‐BCs, *DPP4* was chosen as the research target because of the availability of a selective inhibitor, sitagliptin, which targets DPP4 [25], and because the relationship between DPP4 and MMAE resistance had not been studied yet. Then, by searching TCGA database, we found that, compared with the OS of patients with BC exhibiting low *DPP4* expression, the OS of patients with BC exhibiting high *DPP4* expression was significantly reduced (Fig. 2D). In addition, BC in patients with high *DPP4* expression was categorized as having higher pathological T and N grades and a higher clinical stage (Fig. 2E). qRT‐PCR revealed that compared with parental BC cells, the *DPP4* expression in MR‐BCs increased significantly (Fig. S6, Table S2). Western blotting found that DPP4 protein was also increased in MR‐BCs (Fig. 2F). These results confirmed the results of RNA sequencing.

### 3.3 Knockdown of *DPP4* in MR‐BCs suppressed cell proliferation, migration, and invasion

**Fig. 3:**
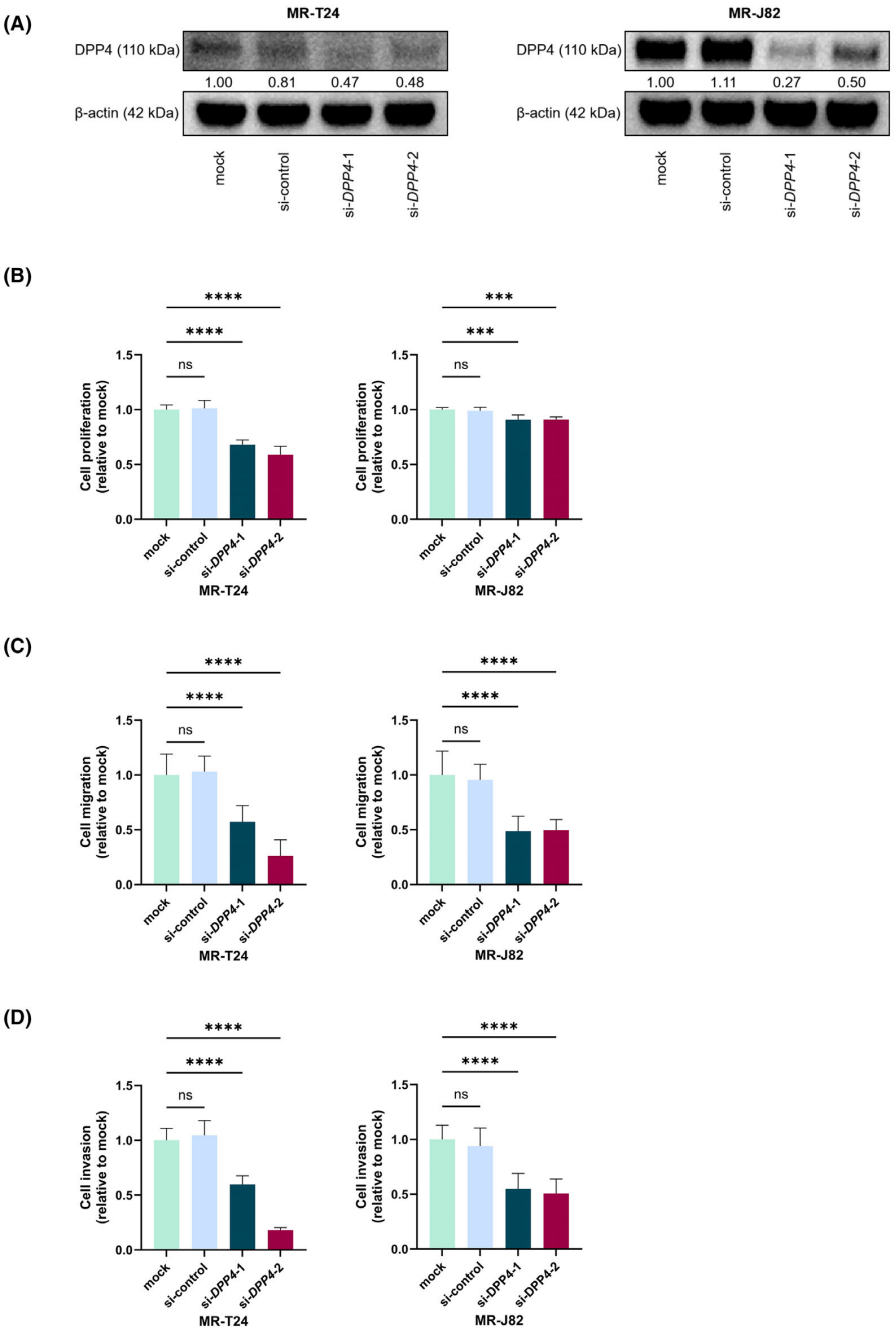
Knockdown of *DPP4* suppressed the viability of MR‐BCs. (A) DPP4 protein levels in MR‐BCs after si‐*DPP4* transfection, as determined by western blotting (*n* = 3). (B) Cell proliferation according to XTT assay after si‐*DPP4* transfection (*n* = 6). (C) Cell migration according to wound healing assay after si‐*DPP4* transfection (*n* = 3). (D) Cell invasion according to Matrigel invasion assay after si‐*DPP4* transfection (*n* = 8). si‐*DPP4* transfection concentration: 50 nm. The statistical tests used were one‐way ANOVAs (B, C, and D). ns, no significance; ***P < 0.001; ****P < 0.0001. The error bars indicate SD. MR‐J82, MMAE‐resistant J82; MR‐T24, MMAE‐resistant T24.

Currently, research on the relationship between DPP4 and BC is still rare. One study showed that *DPP4* expression was higher in samples from urothelial carcinoma patients with advanced tumor stage, knockdown of *DPP4* in BC cells (J82 and RTCC1 cells) significantly inhibited cell proliferation, migration, and invasion [26]. To investigate the effects of *DPP4* on MR‐BCs, we performed loss‐of‐function assays using siRNAs. The results of qRT‐PCR showed that both siRNAs (si‐*DPP4*‐1 and si‐*DPP4*‐2) significantly reduced *DPP4* mRNA levels (Fig. S7 and Table S3). Western blotting also confirmed this result (Fig. 3A). Notably, the proliferation of si‐*DPP4*‐transfected MR‐BCs was decreased compared with that of the control cells (Fig. 3B). Cell migration and invasion were also both inhibited by *DPP4* knockdown (Fig. 3C,D and Figs S8 and S9). These results indicated that knockdown of DPP4 could suppress the viability of MR‐BCs.

### 3.4 The DPP4 inhibitor sitagliptin effectively inhibited the proliferation, migration, and invasion of parental and MMAE‐resistant BC cell lines

**Fig. 4:**
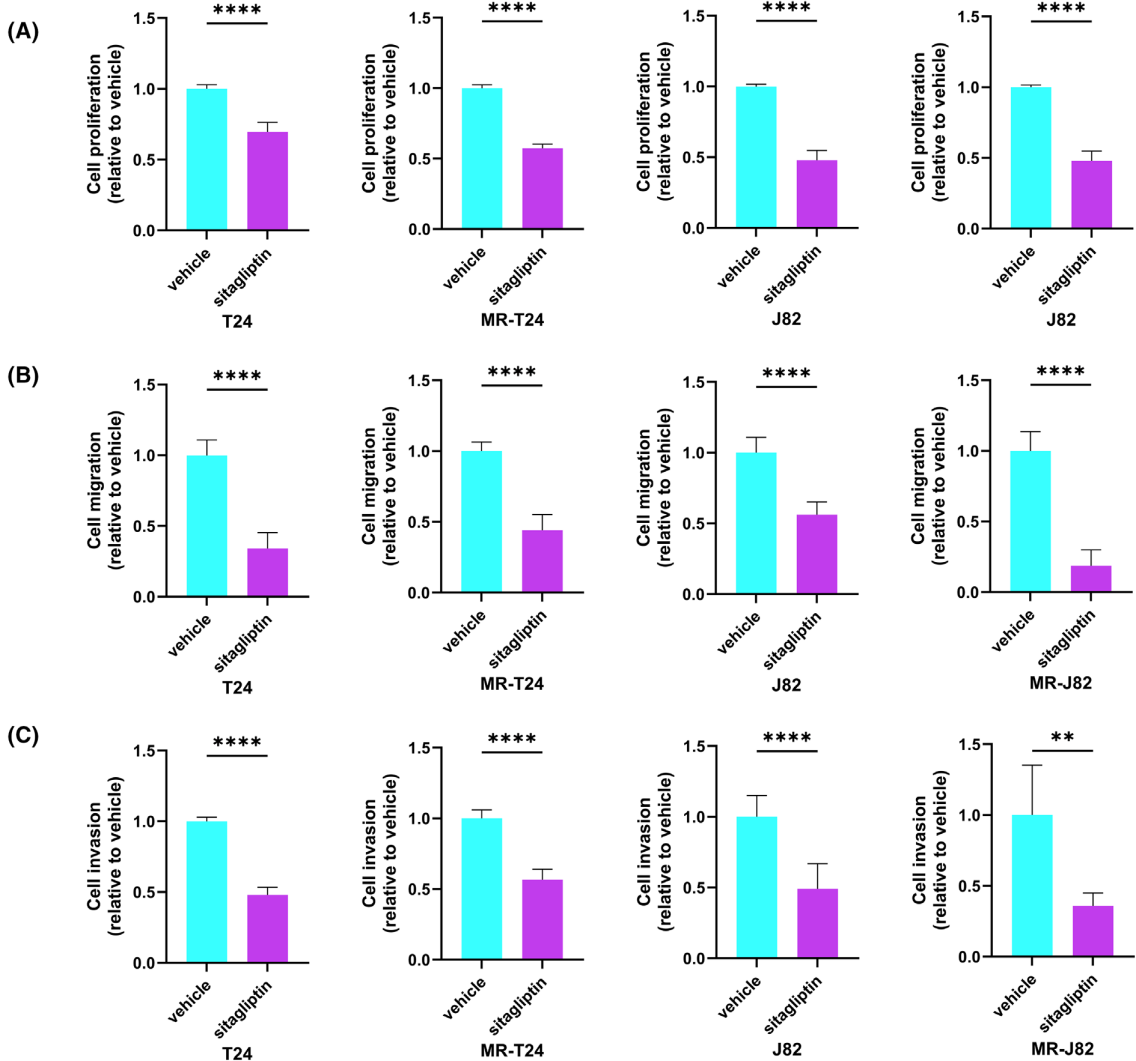
Sitagliptin decreased the viability of parental BC cells and MR‐BCs. (A) Cell proliferation according to XTT assay after sitagliptin treatment (*n* = 8). (B) Cell migration according to wound healing assay after sitagliptin treatment (*n* = 3). (C) Cell invasion according to Matrigel invasion assay after sitagliptin treatment (*n* = 8). Sitagliptin concentration: 1.5 mm. The statistical tests used were unpaired *t*‐tests. ns, no significance; **P < 0.01; ****P < 0.0001. The error bars indicate SD. MR‐J82, MMAE‐resistant J82; MR‐T24, MMAE‐resistant T24.

Sitagliptin, as a DPP4‐specific inhibitor, has been shown to have inhibitory effects on various tumor activities [27, 28, 29, 30, 31]. To investigate the effects of sitagliptin on parental BC cells and MR‐BCs, we first determined the IC_50_ (Fig. S10). The IC_50_ values of the four cell lines for sitagliptin were similar (T24: 1.669 mm, J82: 1.295 mm, MR‐T24: 1.488 mm, MR‐J82: 1.598 mm). Some studies have shown that sitagliptin also has a significant inhibitory effect on other tumors at this concentration [28, 31]. Subsequent loss‐of‐function assays at a sitagliptin concentration of 1.5 mm showed that sitagliptin significantly inhibited cell proliferation (Fig. 4A), cell migration (Fig. 4B and Fig. S11) and cell invasion (Fig. 4C and Fig. S12) in both parental BC cells and MR‐BCs. This indicated that sitagliptin had significant inhibitory effects on both parental and MMAE‐resistant BC cell lines.

### 3.5 Sitagliptin effectively reversed the resistance of MR‐BCs to MMAE by restoring inhibition of the AKT pathway

**Fig. 5:**
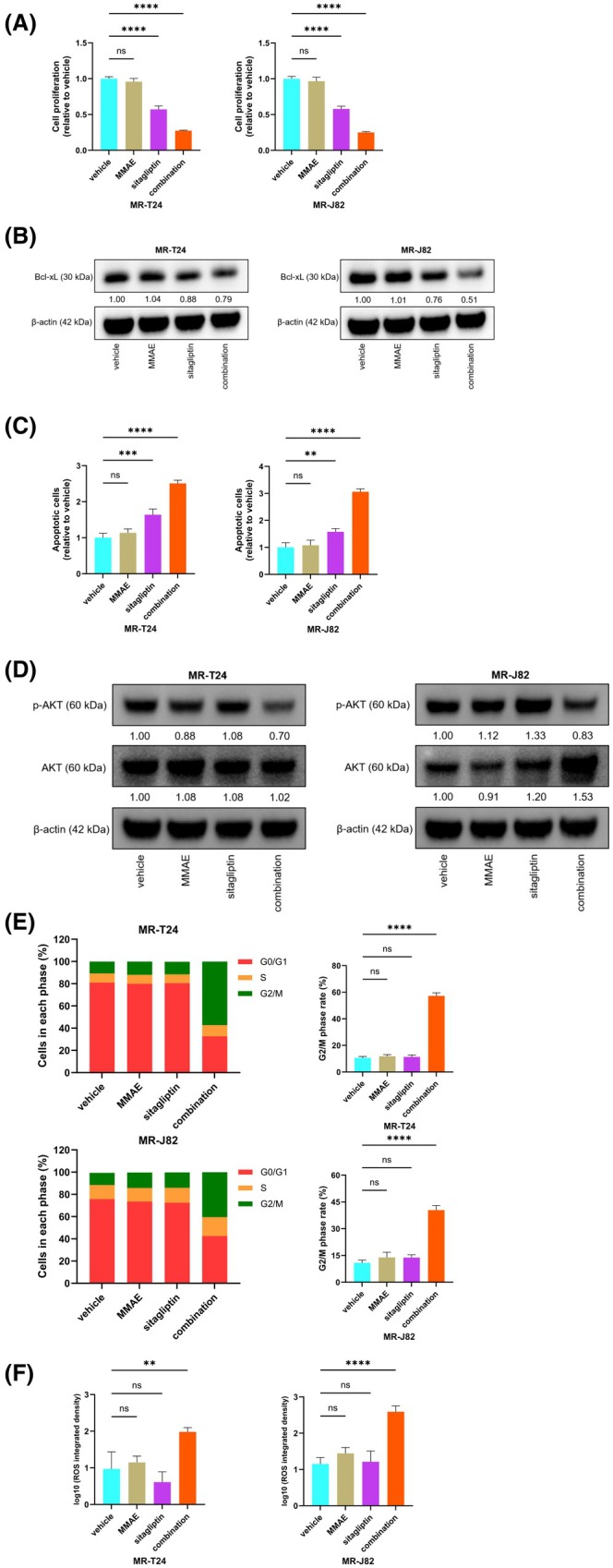
Cotreatment with MMAE and sitagliptin reversed the resistance of MR‐BCs to MMAE. (A) Cell proliferation according to XTT assay after MMAE and/or sitagliptin treatment (*n* = 8). (B) Protein levels of Bcl‐xL in MR‐BCs after MMAE and/or sitagliptin treatment, as determined by western blotting (*n* = 3). (C) Apoptosis assay using flow cytometry after MMAE and/or sitagliptin treatment (*n* = 3). (D) Protein levels of phospho‐AKT and AKT in MR‐BCs after MMAE and/or sitagliptin treatment, as determined by western blotting (*n* = 3). (E) Cell cycle assay using flow cytometry after MMAE and/or sitagliptin treatment (*n* = 3). (F) ROS assay of MR‐BCs after MMAE and/or sitagliptin treatment (*n* = 3). MMAE concentration: 30 nm; sitagliptin concentration: 1.5 mm. The statistical tests used were one‐way ANOVAs (A, C, E, and F). ns, no significance; **P < 0.01; ***P < 0.001; ****P < 0.0001. The error bars indicate SD. MMAE, monomethyl auristatin E; MR‐J82, MMAE‐resistant J82; MR‐T24, MMAE‐resistant T24; ROS, reactive oxygen species.

Cotreatment with sitagliptin and paclitaxel has been shown to enhance the apoptotic effects of paclitaxel on ovarian cancer cells [32]. Given that paclitaxel is a taxane drug [33], we investigated the effects of cotreatment with MMAE and sitagliptin on MMAE‐resistant cells. Interestingly, the combination of MMAE and sitagliptin significantly suppressed the proliferation ability of MR‐BCs (Fig. 5A) and restored the inhibitory effects on parental cell proliferation at the same MMAE concentration (Fig. S13). In addition, when the drug concentrations were reduced to 60% of the original, MMAE and sitagliptin still had a strong synergistic effect (Fig. S14). Western blotting indicated that MMAE inhibited the expression of the anti‐apoptotic protein Bcl‐xL in parental BC cells (Fig. S15). Moreover, in MR‐BCs, although MMAE alone had little effect on Bcl‐xL, cotreatment with MMAE and sitagliptin significantly reduced Bcl‐xL expression (Fig. 5B). Apoptosis assays showed that in MR‐BCs, the combination of MMAE and sitagliptin significantly induced apoptosis compared with sitagliptin alone (Fig. 5C and Fig. S16). The above results indicated that sitagliptin could reverse the resistance of MR‐BCs to MMAE.

The AKT pathway is closely related to drug resistance in cancer [34], and MMAE has been shown to be associated with the phosphorylation of AKT [35]. Therefore, we investigated the relationship between the combination treatment and the AKT pathway. Western blotting showed that MMAE effectively inhibited the phosphorylation of AKT in parental BC cells (Fig. S17). In MR‐BCs, the decrease in AKT phosphorylation was reduced by MMAE treatment alone compared with that in parental BC cells; however, cotreatment with sitagliptin and MMAE effectively reversed this effect (Fig. 5D). Cell cycle assays also confirmed that cotreatment significantly arrested MR‐BCs in the G_2_/M phase (Fig. 5E and Fig. S18).

Some studies have shown that ROS production in the mitochondria increases when the AKT pathway is inhibited [36, 37]. Therefore, we examined ROS levels in response to MMAE or MMAE and sitagliptin treatment. ROS assays indicated that compared with ROS levels in parental BC cells (Figs S19 and S20), ROS levels in MR‐BCs did not change in response to MMAE alone (Fig. 5F and Fig. S21) but were significantly increased in response to the combination of MMAE and sitagliptin.

In summary, reactivation of MMAE by sitagliptin inhibited the AKT pathway, and this response may represent a potential mechanism for the reversal of MMAE resistance in MR‐BCs.

### 3.6 Cotreatment with MMAE and sitagliptin effectively inhibited the growth of MMAE‐resistant cells in a xenograft mouse model

To investigate the therapeutic effects of MMAE and sitagliptin cotreatment *in vivo*, we established a xenograft mouse model. In a previous study, the high *in vivo* toxicity of free MMAE resulted in rapid death of BALB/c mice following intraperitoneal injection of MMAE at a concentration of 0.25 mg·kg^−1^ [38]. By contrast, intravenous injection of MMAE at a concentration of 0.1 mg·kg^−1^ did not affect the survival of NOD/SCID mice while allowing mice to maintain a stable body weight and inhibiting the growth of LNCaP prostate cancer tumors [39]. Therefore, we chose to use MMAE at a concentration of 0.1 mg·kg^−1^ in this experiment.

**Fig. 6:**
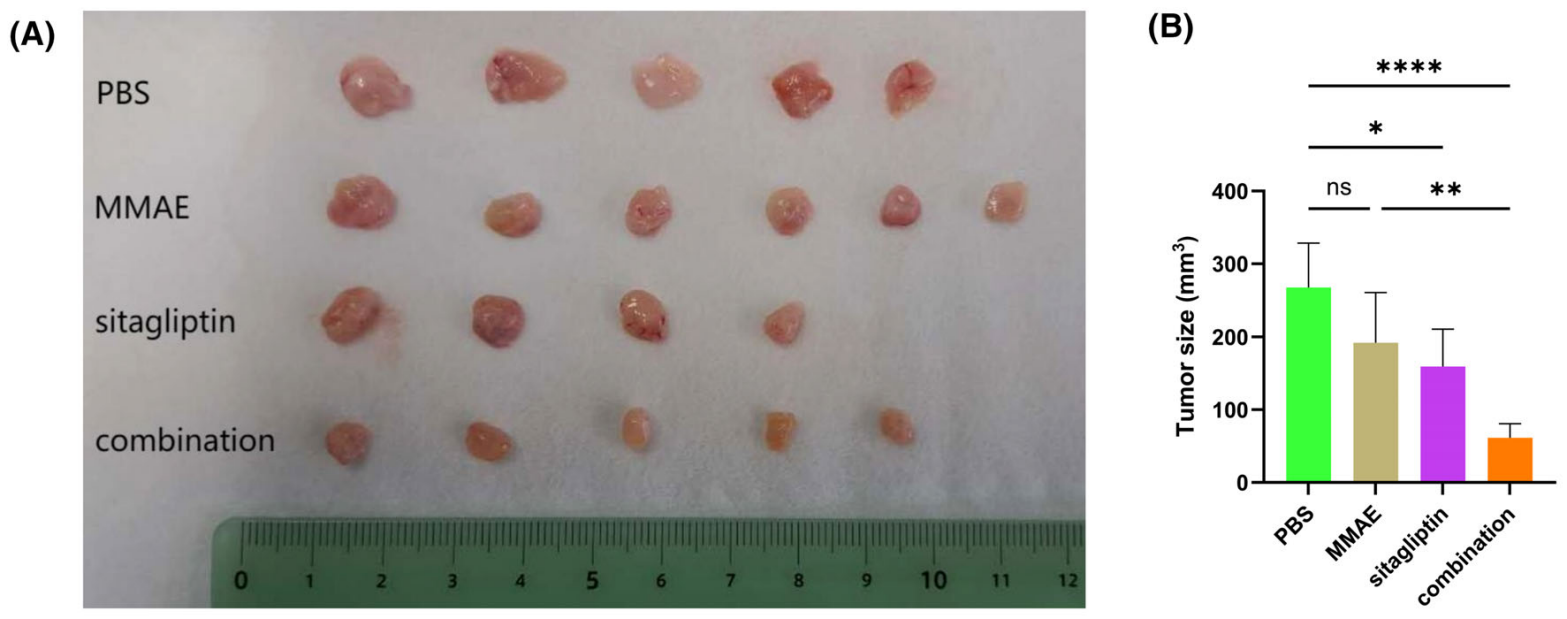
Cotreatment with MMAE and sitagliptin significantly decreased tumor growth in an MR‐T24 xenograft model. (A) Photographs of the tumors. The mice were divided into four groups: PBS, MMAE (0.1 mg·kg^−1^), sitagliptin (200 mg·kg^−1^), and MMAE (0.1 mg·kg^−1^) + sitagliptin (200 mg·kg^−1^). (B) Comparison of tumor volumes among groups (*n* = 6). The statistical tests used was a one‐way ANOVA. ns, no significance; *P < 0.05; **P < 0.01; ****P < 0.0001. The error bars indicate SD. MMAE, monomethyl auristatin E.

At 14 days after inoculation with MMAE‐resistant cells (MR‐T24 cells), nude mice were randomly divided into four groups: PBS control group, the MMAE group, the sitagliptin group, and the combination (MMAE + sitagliptin) group. The treatments were performed, and the mice were sacrificed at 33 days after establishment of xenografts (Fig. 6A). The results showed that sitagliptin effectively inhibited tumor growth in nude mice. Cotreatment with MMAE and sitagliptin had a significant inhibitory effect on the growth of tumor cells (Fig. 6B). There were no significant changes in body weights between groups (Fig. S22).

## 4 Discussion

Currently, research on MMAE resistance has mainly focused on multidrug resistance protein 1 (MDR1), an ATP‐binding cassette transporter. One study revealed that compared with parental cells, MMAE‐resistant Hodgkin lymphoma cells showed increased mRNA and protein expression of *MDR1*. Moreover, inhibiting MDR1 using competitive inhibitors significantly increases the MMAE concentration in resistant Hodgkin lymphoma cells, improves drug sensitivity, and reduces the IC_50_ [40]. In this study, although there was no significant difference (*P* > 0.05), RNA sequencing showed that the FPKM of *ABCB1* (*MDR1*) in MR‐BCs was increased relative to that in the parental cells (T24: 0.02; MR‐T24: 51.85; J82: 1.00; MR‐J82: 89.96), suggesting that our MR‐BCs were suitable as resistant cells.

DPP4 is a cell‐surface protease belonging to the prolyloligopeptidase family. It is a transmembrane glycoprotein composed of 766 amino acids, with a relative molecular weight of 110 kDa [41, 42]. DPP4 exerts various biological functions, with roles in glucose homeostasis, inflammation, immune regulation, cell adhesion, migration, invasion, differentiation, and apoptosis [41, 43]. Moreover, *DPP4* is also expressed in body fluids, including the plasma and serum, in the form of soluble DPP4 (sDPP4/sCD26). sDPP4 is involved in regulating some physiological functions, including endothelial dysfunction and immune regulation, and is used as a potential biomarker for some diseases [42, 44]. By cleaving cytokines and chemokines, DPP4 can act as both a tumor activator and tumor suppressor, suggesting potential as a target for tumor therapy drugs [43, 44]. In addition, the level of sDPP4/sCD26 protein is considered a potential biomarker for the screening, monitoring and prognosis of some cancers [44, 45].

The relationship between DPP4 and MMAE resistance has not yet been studied, and our current findings are the first to highlight DPP4 as a potential modulator of MMAE resistance. By investigating the TCGA database, we found that high *DPP4* expression was related to a lower OS rate, higher pathological T and N grades, and a higher pathological stage in patients with BC when compared with low *DPP4* expression. The reason why there was no significant difference in the pathological M grade may be due to the small sample size available. Therefore, DPP4 could be a potential biomarker for predicting BC prognosis and MMAE resistance. One study showed that *DPP4* expression is increased in sunitinib‐resistant renal cell carcinoma, whereas DPP4 inhibition enhances sunitinib efficacy [46]. *DPP4* expression is also higher in methotrexate‐resistant choriocarcinoma, and knockdown of *DPP4* reduces this resistance [47]. Another study demonstrated that DPP4 is significantly upregulated in residual tumor tissue from lung cancer patients treated with osimertinib compared with the tumor tissues from patients who did not receive osimertinib [48]. These experimental results support our research findings on the role of DPP4 in acquired resistance.

Sitagliptin is a potent inhibitor of DPP4 and is used to treat type 2 diabetes. Sitagliptin increases the release of glucagon‐like peptide 1 and glucose‐dependent insulin tropic polypeptide, thus stimulating insulin secretion and reducing glucagon levels [25]. Notably, hyperglycemia is one of the main adverse events in patients receiving EV treatment [49]. This may be related to the increased expression of DPP4 observed in this study. Although sitagliptin exhibits some off‐target effects, it is still regarded as a safe drug. One study found that the off‐target inhibition of selective DPP‐4 inhibitors is associated with immune dysfunction, impaired healing, and skin reactions. However, the study also indicated that sitagliptin is well‐tolerated, with a low incidence of hypoglycemia, and can be used to treat diabetic patients with renal or hepatic dysfunction [50]. Another study showed that DPP4 inhibitors have the potential for off‐target cardiovascular effects, but does not increase blood pressure [51]. To date, studies have found that sitagliptin can inhibit the viability of some tumors and synergistically increase the efficacy of chemotherapy drugs. For example, Shin et al. revealed that sitagliptin reduces the activity of colorectal cancer and enhances the anticancer effects of 5‐fluorouracil [30]. You et al. also confirmed that sitagliptin inhibits glioma cell proliferation, induces apoptosis, and enhances temozolomide cytotoxicity [31].

As a member of the Bcl‐2 family, Bcl‐xL is an anti‐apoptotic protein that is downregulated when apoptosis increases [52]. Our study showed that sitagliptin reduced the expression of Bcl‐xL in MR‐BCs and that the expression of Bcl‐xL was even lower when sitagliptin was combined with MMAE. Additionally, combination therapy significantly increased apoptosis, and our xenograft model indicated that sitagliptin inhibited the growth of MMAE‐resistant tumors, with cotreatment enhancing these growth inhibitory effects. The above results indicate that the resistance of MR‐BCs to MMAE was reversed by sitagliptin.

Because the AKT pathway serves as a key link in regulating the multidrug resistance of cancers [34], we investigated the relationship between the reversal of MMAE resistance and the AKT pathway. Treatment with sitagliptin alone had no effect on phospho‐AKT levels, whereas the combination of sitagliptin and MMAE significantly reduced the levels of phospho‐AKT. Thus, these results showed that cotreatment inhibited the AKT pathway and reversed drug resistance, consistent with other studies. Indeed, Zeng et al. found that combination treatment with Cel‐CSO/taxol nanoparticles significantly increased the apoptosis rate and reversed the drug resistance of breast cancer cells to taxol by inhibiting the AKT pathway [53]. Inhibiting the activation of the AKT pathway can also reverse the drug resistance of many tumors, including lung cancer [54], hepatocellular carcinoma [55], and BC [56]. ROS are produced by mitochondria in cells, and large amounts of ROS can cause damage to nucleic acids and proteins, leading to apoptosis [57]. Our study revealed that the combination of sitagliptin and MMAE significantly increased the level of ROS. In addition, flow cytometry analysis showed that cotreatment with MMAE and sitagliptin restored the arrest of cells in the G_2_/M phase. Therefore, cotreatment with MMAE and sitagliptin reversed the resistance of MR‐BCs to MMAE by inhibiting the AKT pathway.

This study demonstrated that sitagliptin may reverse the resistance of MR‐BCs to MMAE, indicating the potential feasibility of a combination therapy with MMAE and sitagliptin. This discovery may provide help for chemotherapy, adjuvant therapy, or neoadjuvant therapy in bladder cancer. However, limitations still exist, requiring further investigation to elucidate the underlying mechanisms of their synergistic effects and to validate these findings in clinical research. Because MMAE is expected to play an increasingly important role in the treatment of urothelial carcinoma in the future, the mechanisms of MMAE resistance and the potential to overcome MMAE resistance, as proposed in this study, may provide new strategies for the treatment of BC. Future studies should aim to improve our understanding of the reasons for the resistance of BC to MMAE. The relationship between sDPP4 and BC should also be investigated. In addition, the findings were based only on cell line studies, and it is necessary to confirm whether these findings are also consistent with results from actual clinical samples.

## 5 Conclusion

In this study, we found that *DPP4* expression was significantly increased in MR‐BCs, and we clarified a partial mechanism of BC resistance to MMAE. DPP4 was found to be associated with MMAE resistance in BC cells and was a potential biomarker for assessing the BC malignancy and MMAE resistance. DPP4 inhibition suppressed BC cell viability. Targeting DPP4 may lead to reversal of MMAE resistance in MR‐BCs. These findings provided new strategies for the treatment of BC.

## Conflict of interest

The authors declare no conflict of interest.

## Author contributions

GL, HY, and HE designed the study. GL, SS, MT, JA, and IF acquired the data. GL, ST, HY, TS, RM, YY, and HE analyzed the data. GL, SS, and HY prepared the paper. All authors reviewed the paper.

## Ethics approval and consent to participate

Animal studies were approved by the Kagoshima University Animal Experiment Committee (MD24071), and the experiments were conducted in accordance with the Animal Use Consent Guidelines of the Kagoshima University Animal Care and Use Committee. Clinical data from the study patients were obtained from TCGA, a publicly available cancer genome database, and no individual ethical approval or written informed consent was obtained.

## Supporting information


**Fig. S1.** Image of migration assay in parental and MMAE‐resistant cells after MMAE treatment.
**Fig. S2.** Image of invasion assay in parental and MMAE‐resistant cells after MMAE treatment.
**Fig. S3.** Apoptosis assay in parental and MMAE‐resistant cells after MMAE treatment.
**Fig. S4.** Cell cycle assay in parental and MMAE‐resistant cells after MMAE treatment.
**Fig. S5.** KEGG analysis of 702 upregulated genes in MMAE‐resistant cells.
**Fig. S6.** *DPP4* mRNA levels in parental and MMAE‐resistant cells.
**Fig. S7.** *DPP4* mRNA levels in MMAE‐resistant cells after si‐*DPP4* transfection.
**Fig. S8.** Image of migration assay in MMAE‐resistant cells after si‐*DPP4* transfection.
**Fig. S9.** Image of invasion assay in MMAE‐resistant cells after si‐*DPP4* transfection.
**Fig. S10.** IC_50_ values of parental and MMAE‐resistant cells treated with sitagliptin.
**Fig. S11.** Image of migration assay in parental and MMAE‐resistant cells after sitagliptin treatment.
**Fig. S12.** Image of invasion assay in parental and MMAE‐resistant cells after sitagliptin treatment.
**Fig. S13.** Cell proliferation according to XTT assay of parental cells after MMAE treatment.
**Fig. S14.** Cell proliferation according to XTT assay of MMAE‐resistant cells after MMAE and sitagliptin treatment.
**Fig. S15.** Western blotting of Bcl‐xL in parental cells after MMAE treatment.
**Fig. S16.** Apoptosis assay in MMAE‐resistant cells after MMAE and/or sitagliptin treatment.
**Fig. S17.** Western blotting of phospho‐AKT and AKT in parental cells after MMAE treatment.
**Fig. S18.** Cell cycle assay in MMAE‐resistant cells after MMAE and/or sitagliptin treatment.
**Fig. S19.** ROS assay of MMAE‐resistant cells after MMAE treatment.
**Fig. S20.** Image of ROS assay in parental cells after MMAE treatment.
**Fig. S21.** Image of ROS assay in MMAE‐resistant cells after MMAE and/or sitagliptin treatment.
**Fig. S22.** Body weight changes in mice treated with MMAE and/or sitagliptin.

**Table S1.** Differentially expressed genes of MMAE‐resistant cells versus parental cells.

**Table S2.** qRT‐PCR data of *DPP4* expression in parental and MMAE‐resistant cells.

**Table S3.** qRT‐PCR data of *DPP4* expression in MMAE‐resistant cells after si‐*DPP4* transfection.

## Data Availability

All data necessary to support the conclusions of this study are provided in the article or the Supporting Information. Furthermore, the RNA sequencing raw data of this study are available in the Gene Expression Omnibus (GEO) database under accession number GSE306410.
