# Supplementary material for: Therapeutic strategies for MMAE‐resistant bladder cancer through DPP4 inhibition
Source: Mol Oncol. 2025 Dec 21;20(5):1347–63. doi: 10.1002/1878-0261.70187 (PMC13155154; doi:10.1002/1878-0261.70187)

Supplementary Figure 1

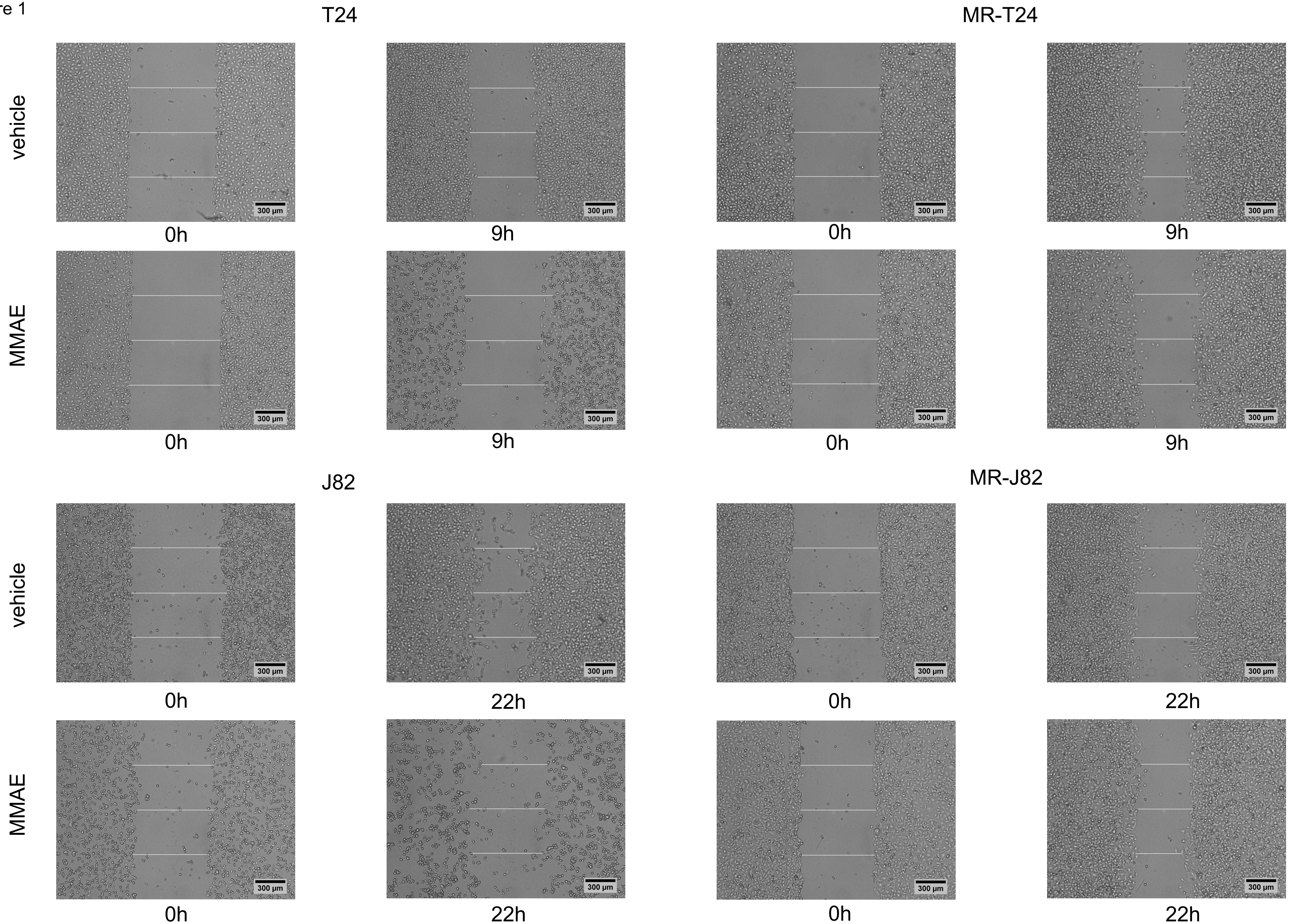

Supplementary Figure 2

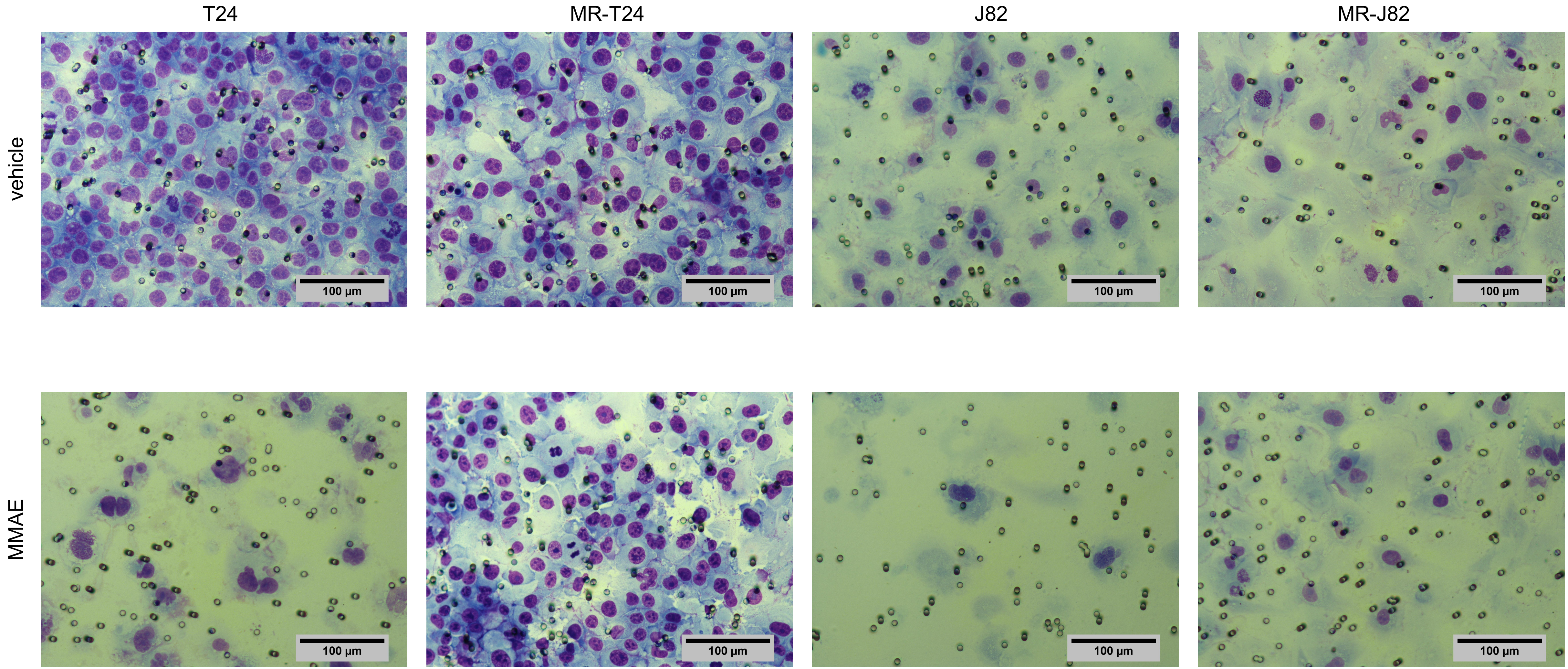

Supplementary Figure 3

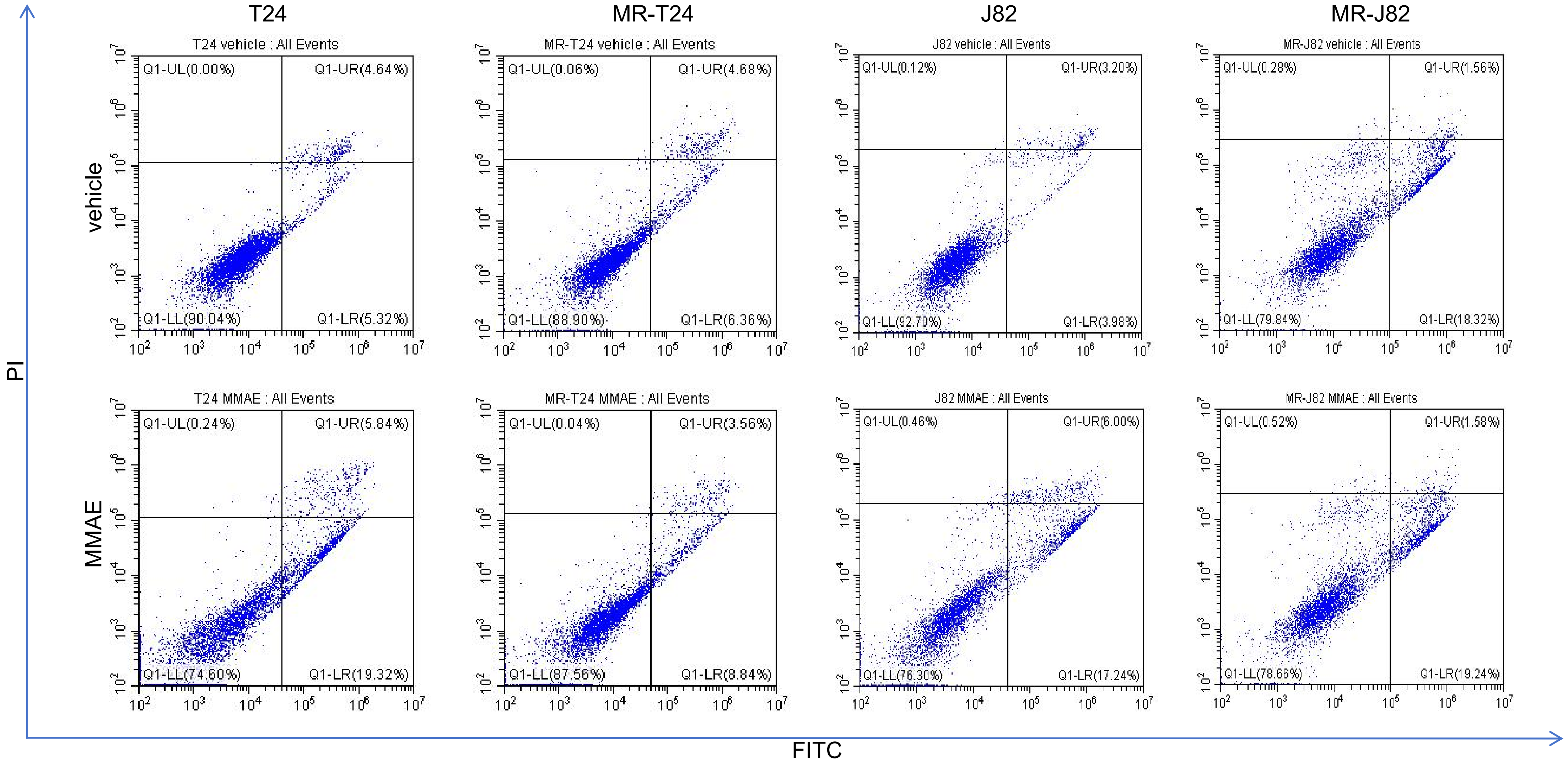

Supplementary Figure 4

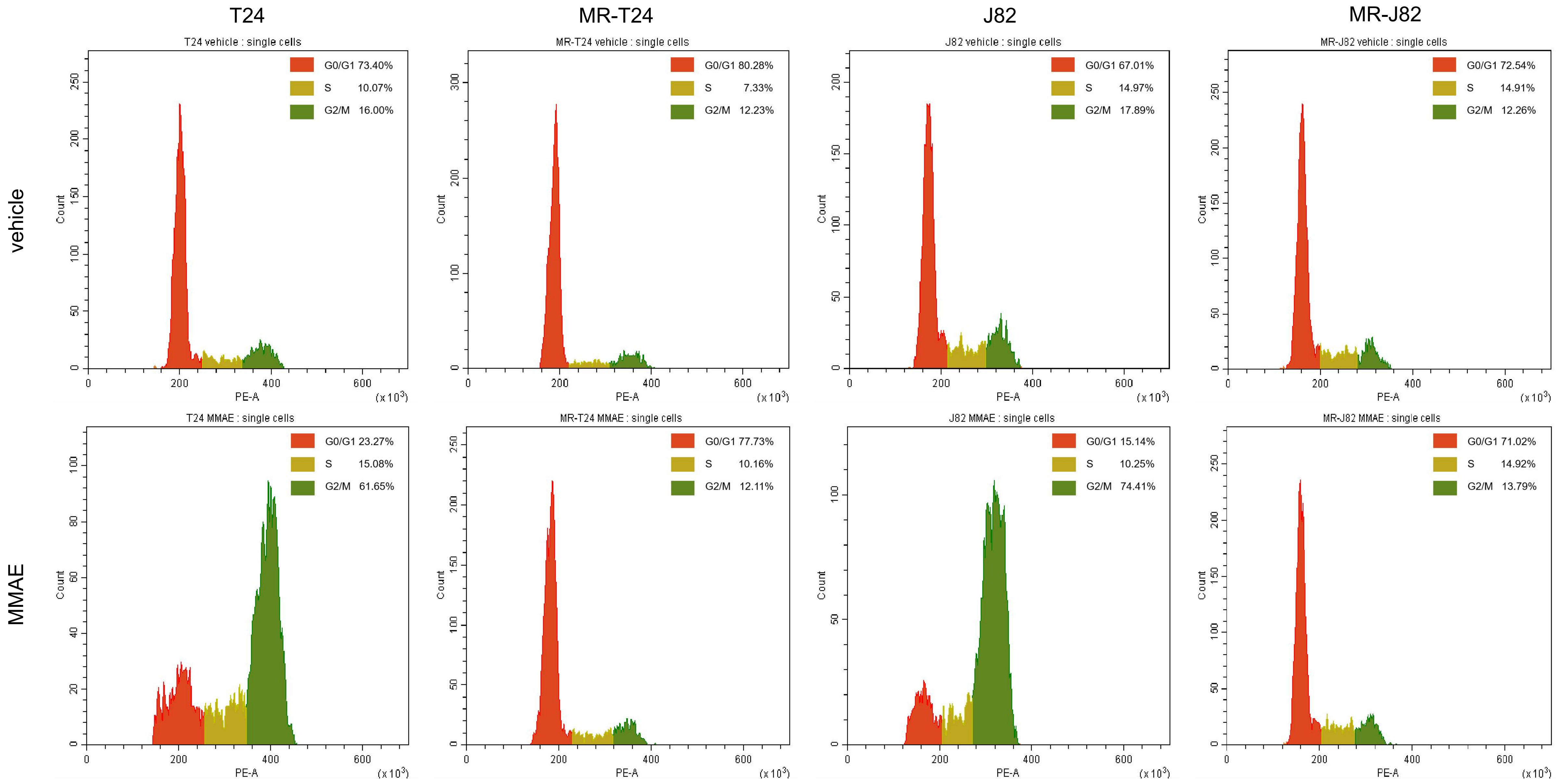

Supplementary Figure 5

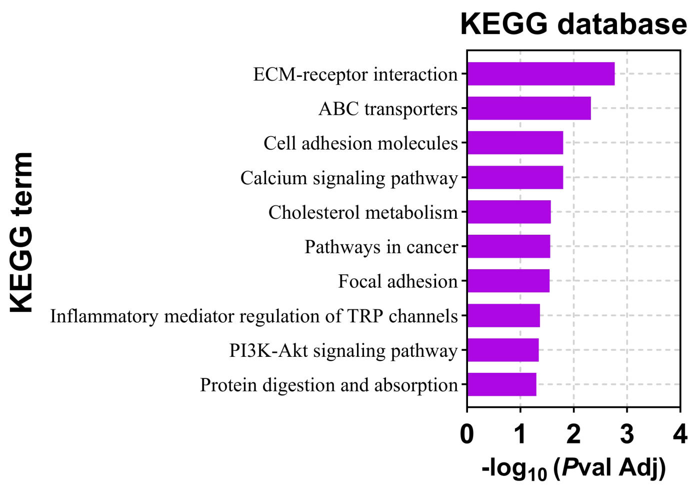

Supplementary Figure 6

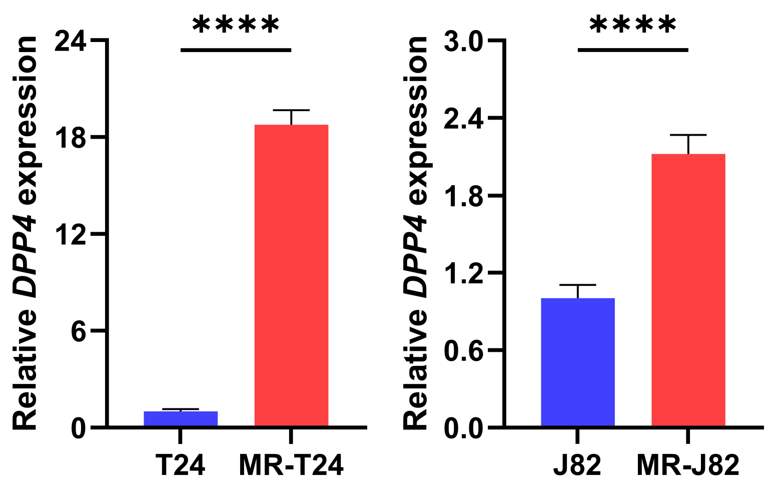

Supplementary Figure 7

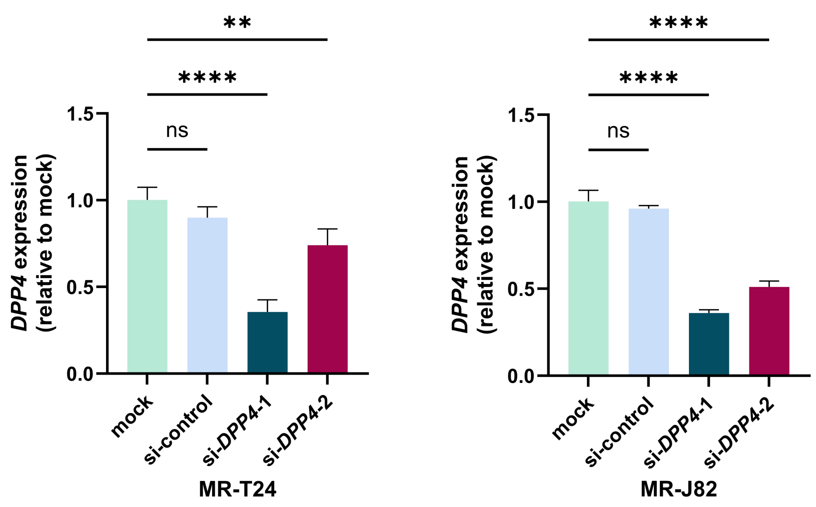

Supplementary Figure 8

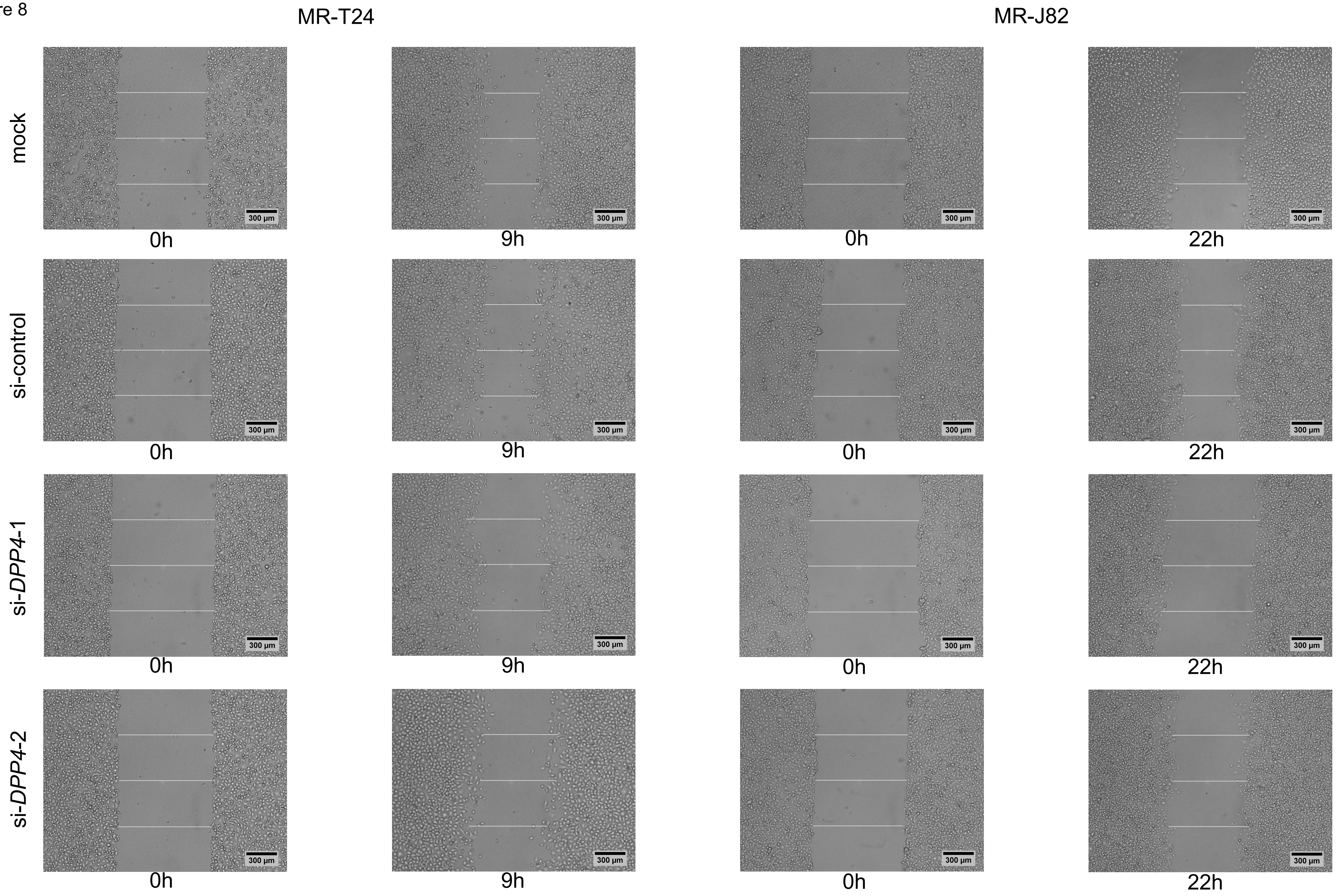

Supplementary Figure 9

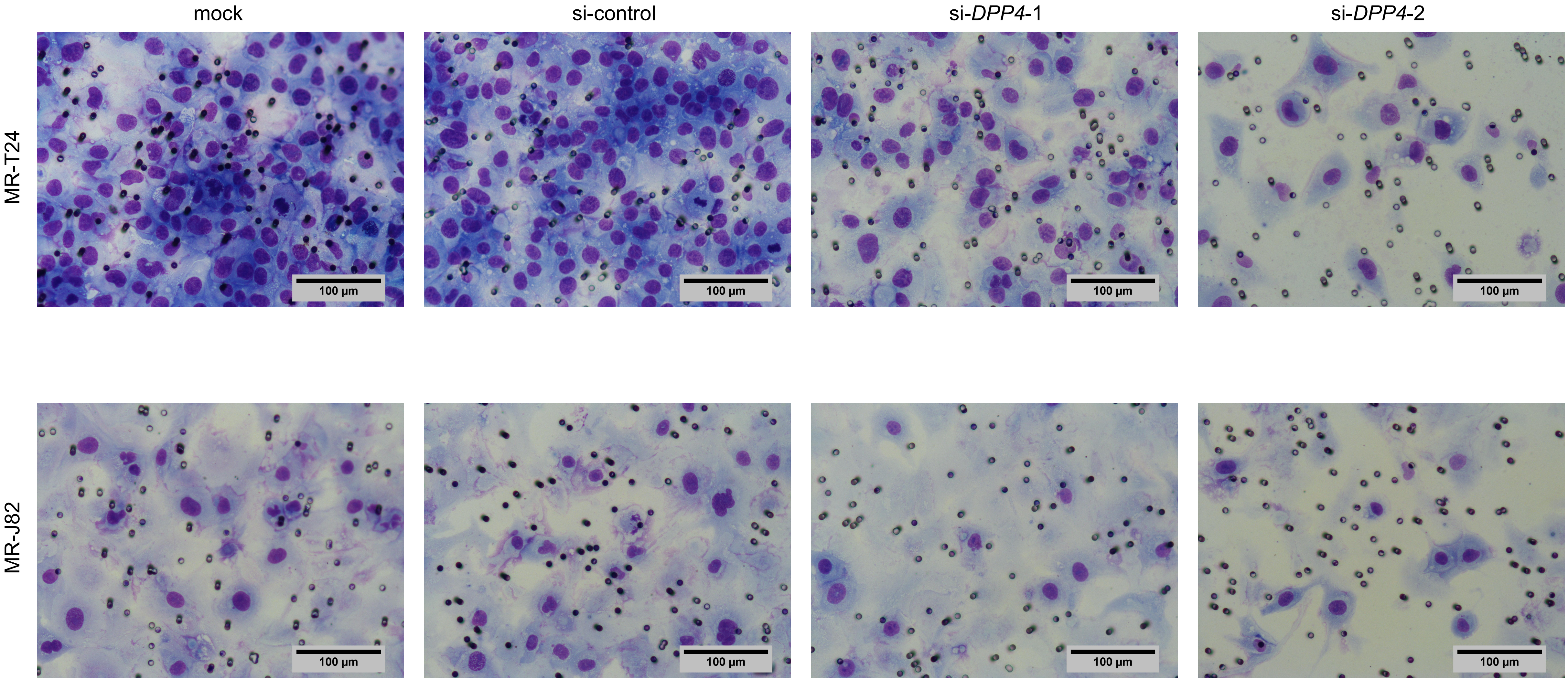

Supplementary Figure 10

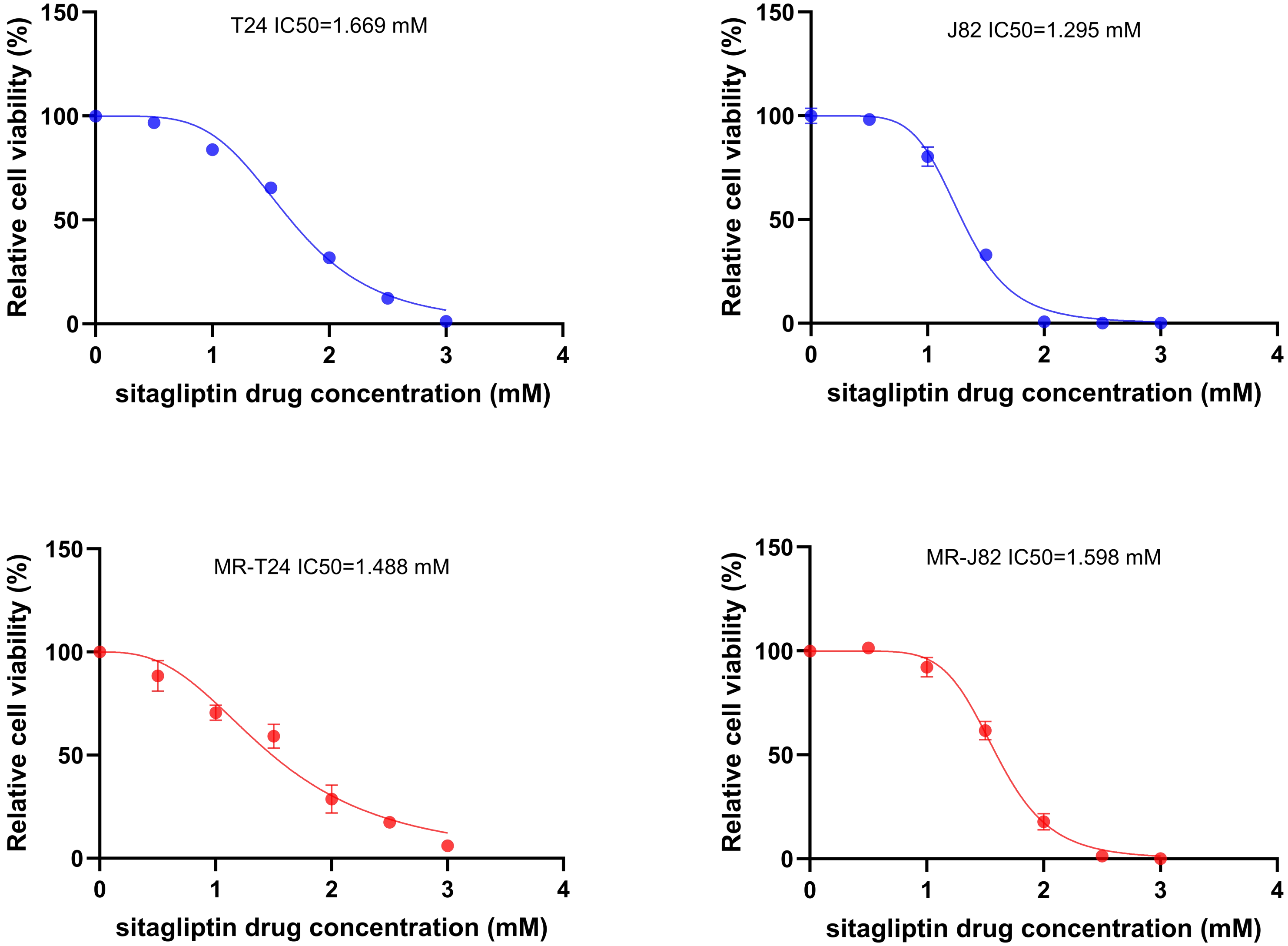

Supplementary Figure 11

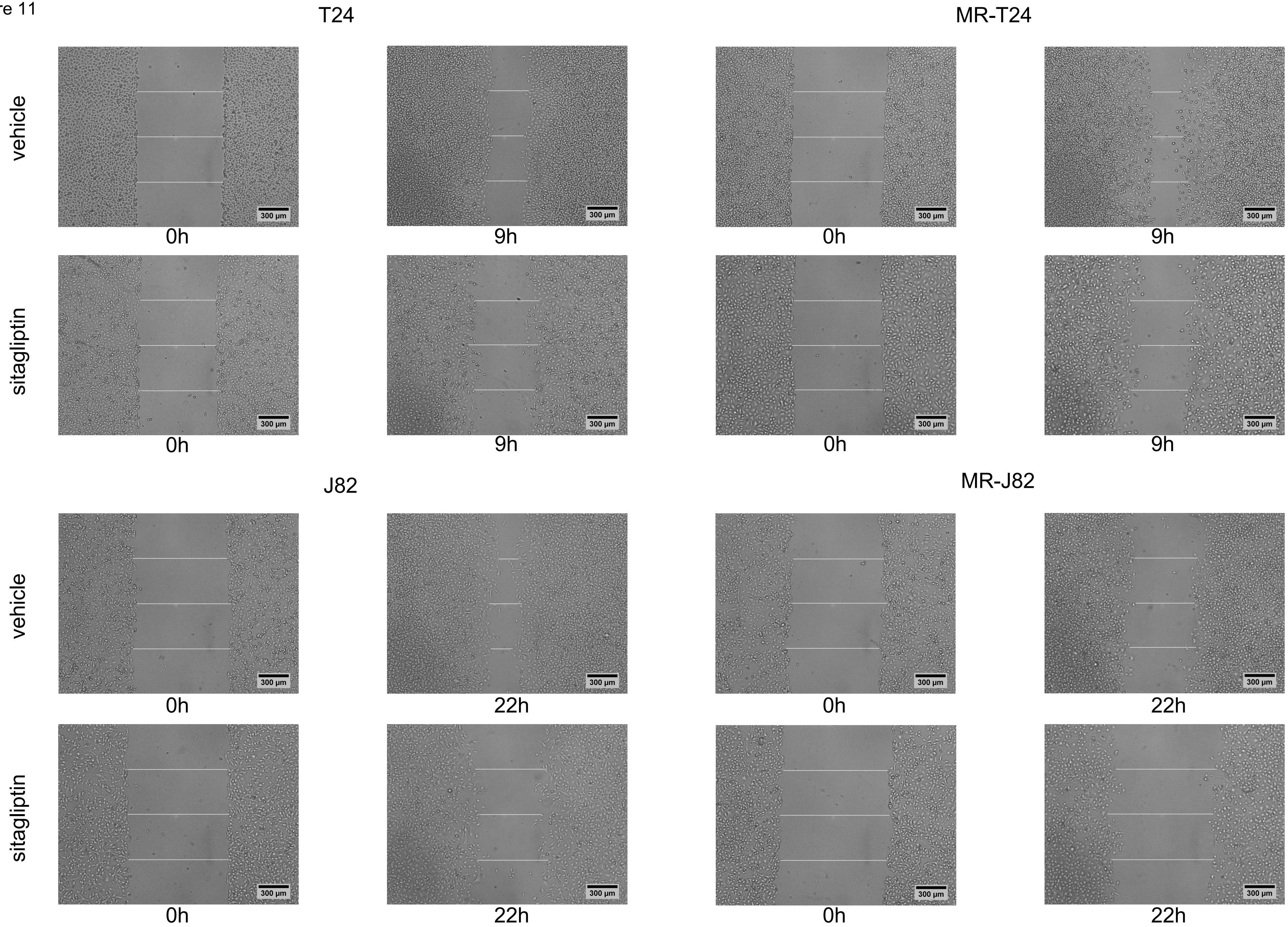

Supplementary Figure 12

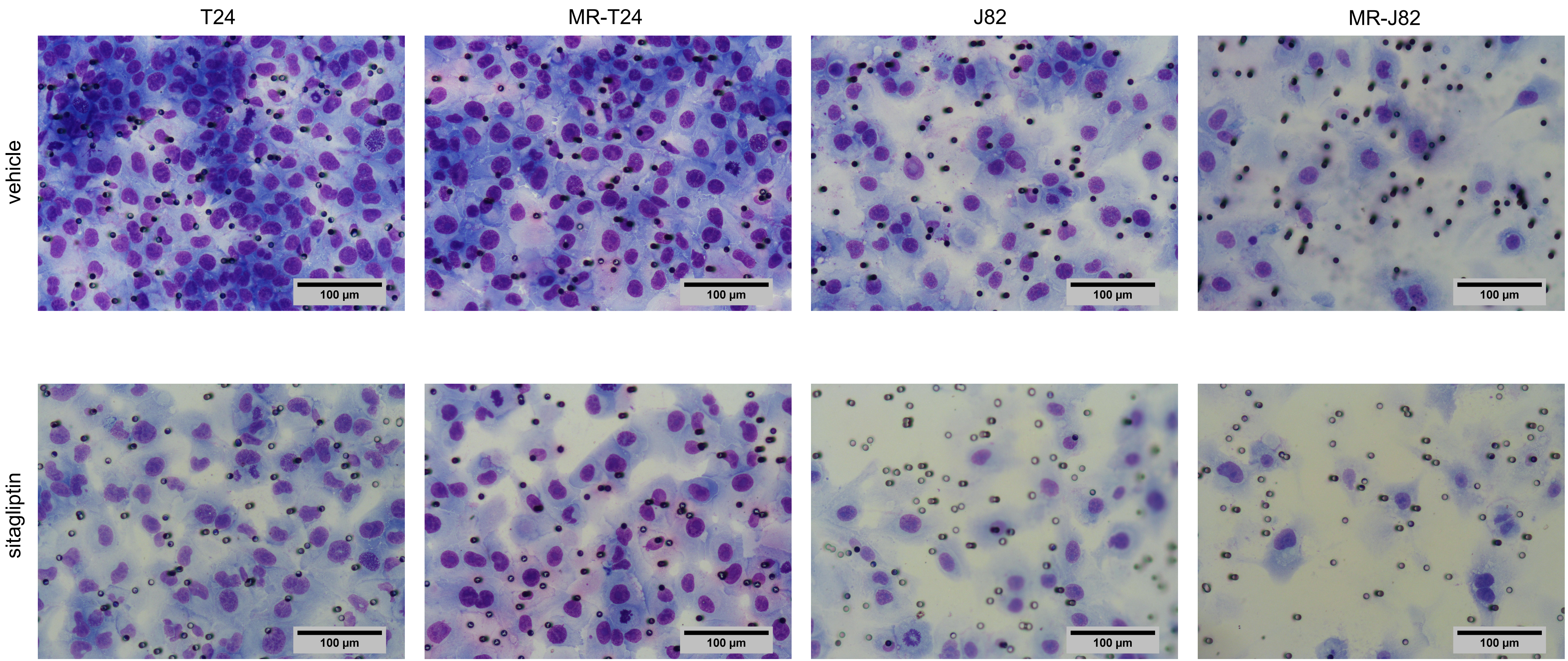

Supplementary Figure 13

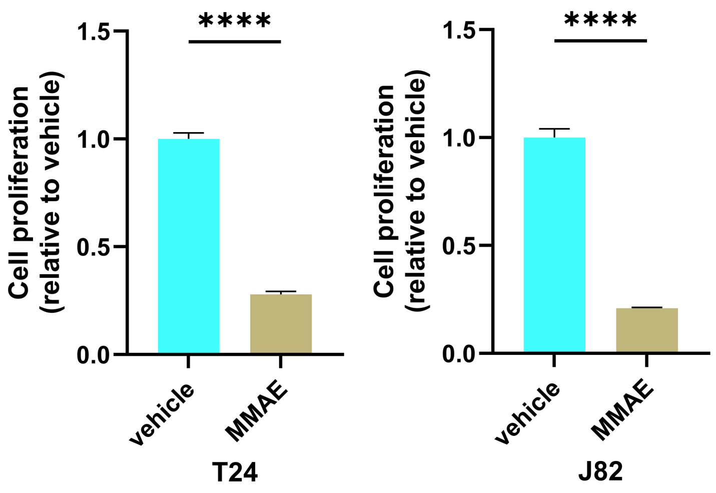

Supplementary Figure 14

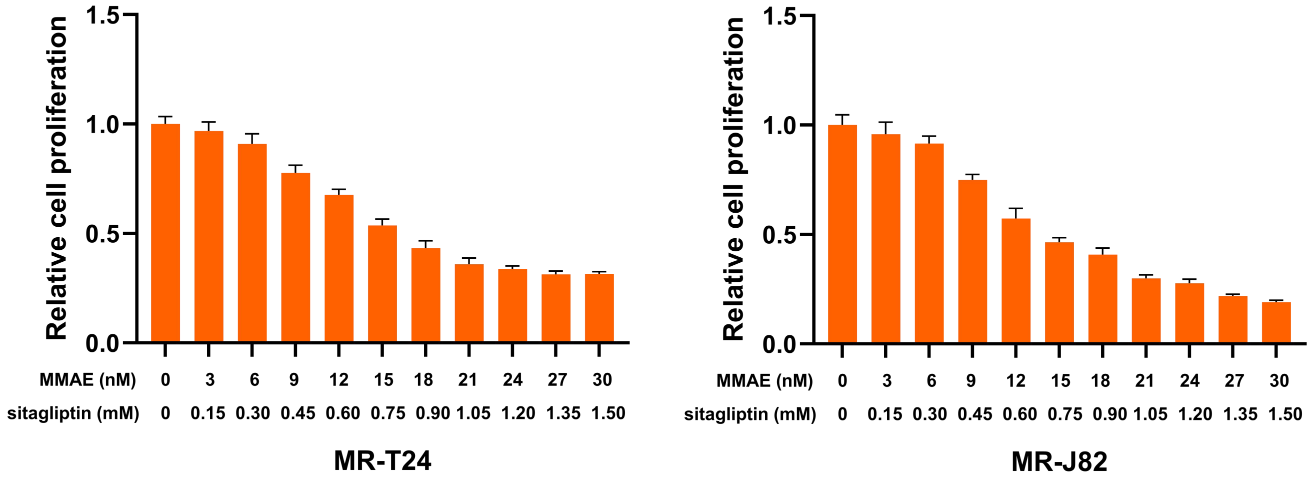

Supplementary Figure 15

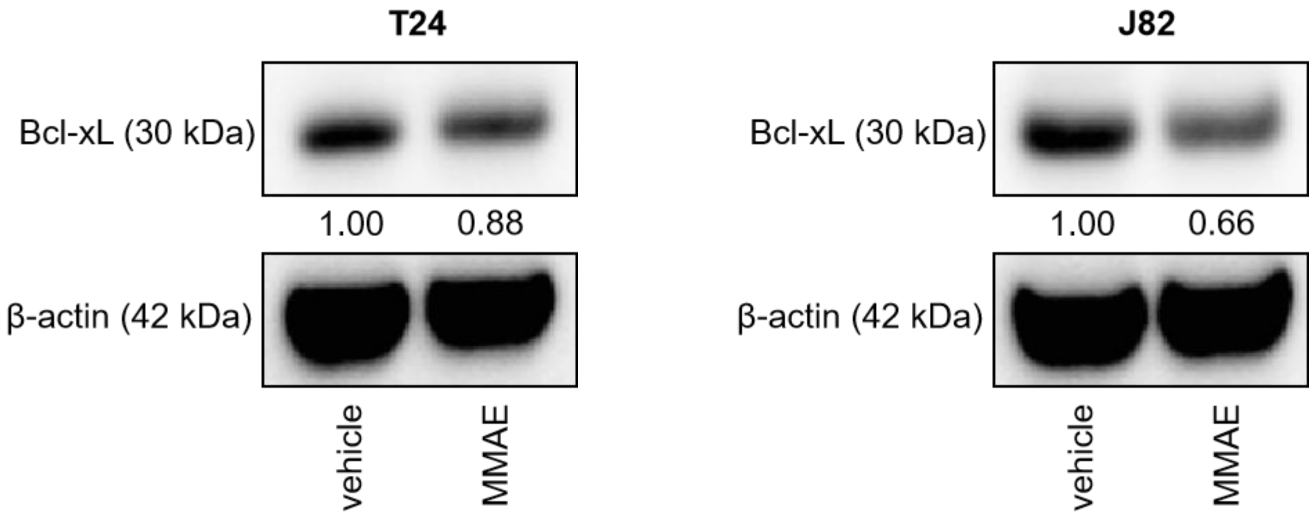

Supplementary Figure 16

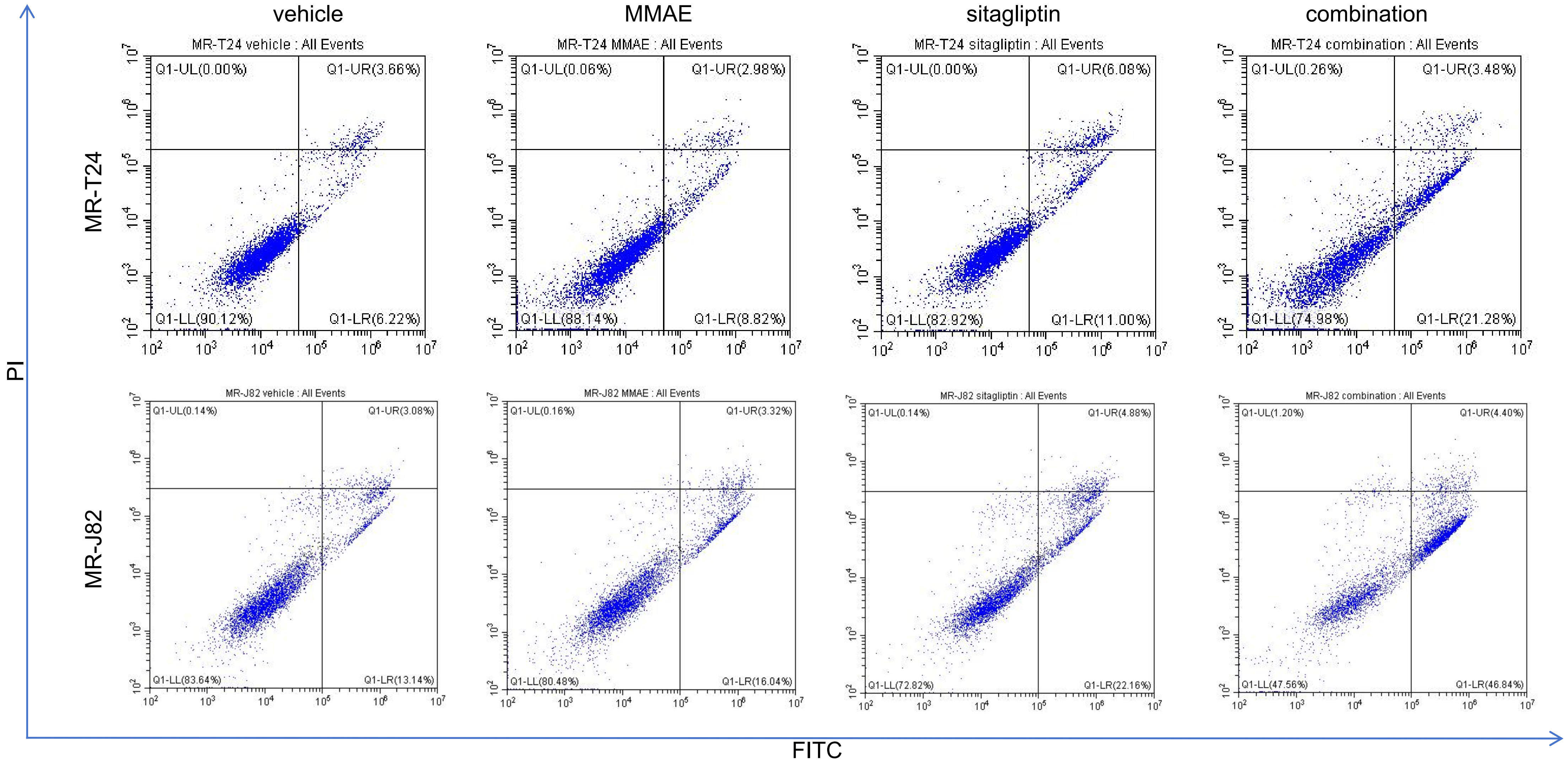

Supplementary Figure 17

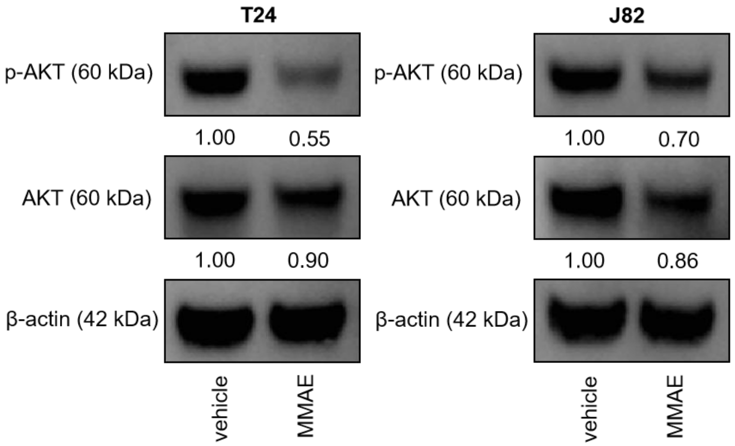

Supplementary Figure 18

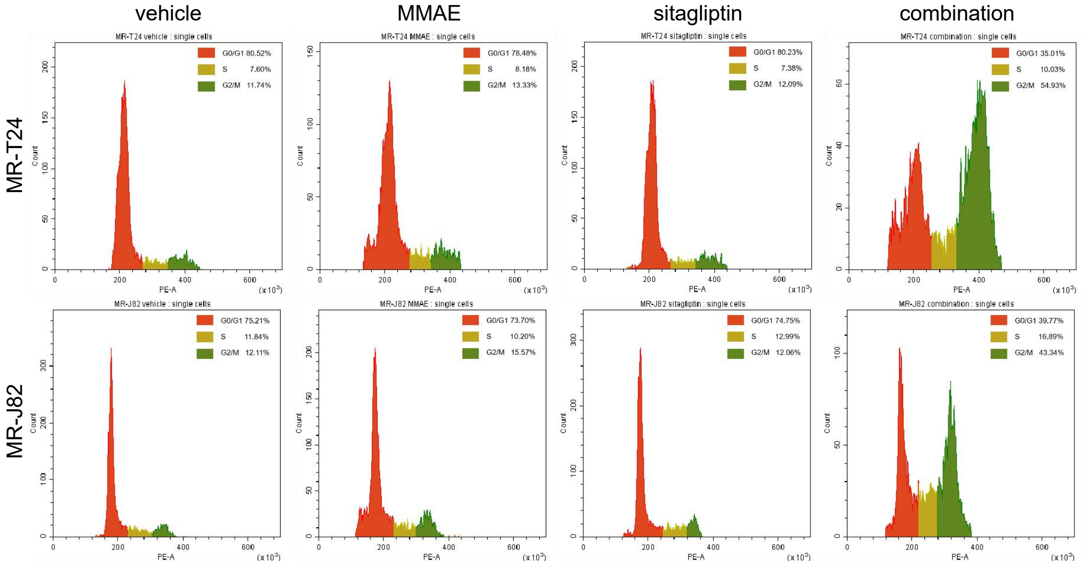

Supplementary Figure 19

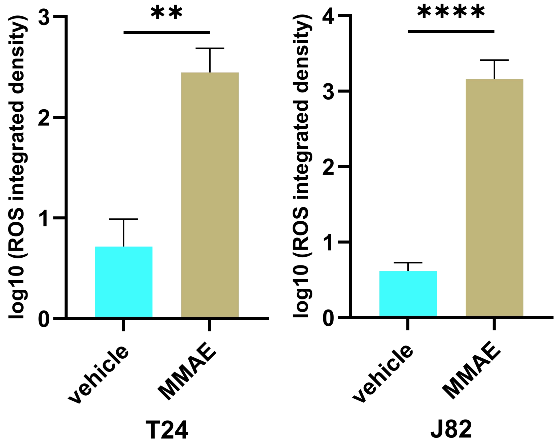

Supplementary Figure 20

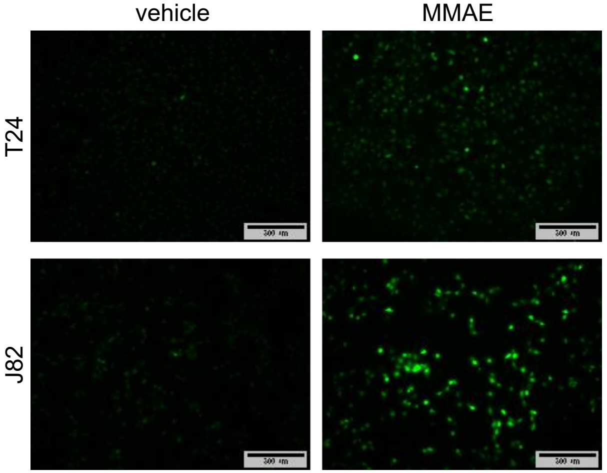

Supplementary Figure 21

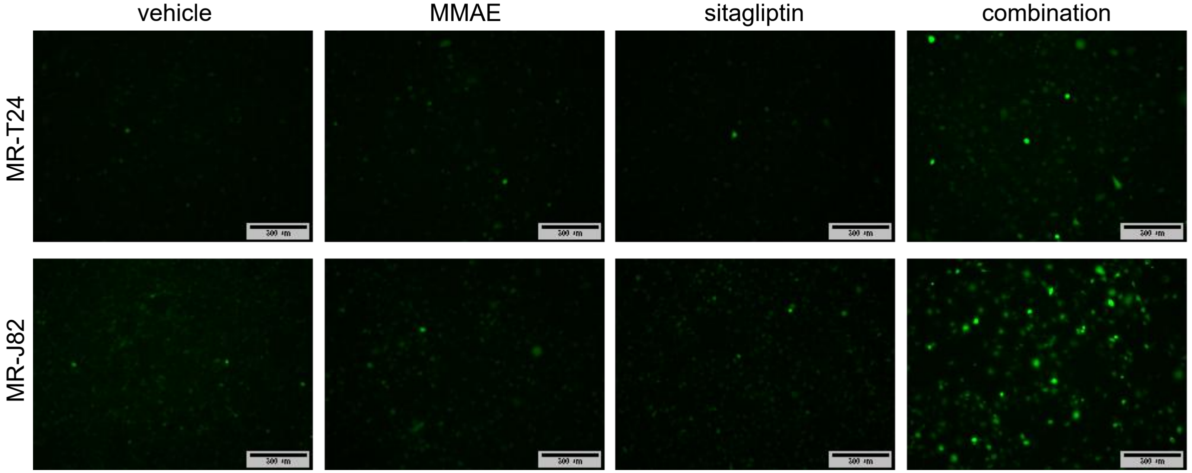

Supplementary Figure 22

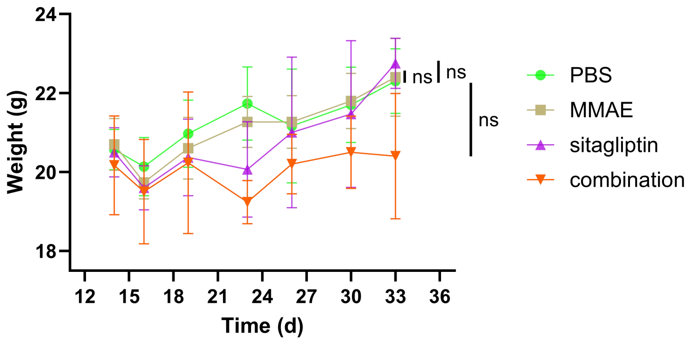

Supplement: Supplementary file 1 — Fig. S1. Image of migration assay in parental and MMAE‐resistant cells after MMAE treatment. Fig. S2. Image of invasion assay in parental and MMAE‐resistant cells after MMAE treatment. Fig. S3. Apoptosis assay in parental and MMAE‐resistant cells after MMAE treatment. Fig. S4. Cell cycle assay in parental and MMAE‐resistant cells after MMAE treatment. Fig. S5. KEGG analysis of 702 upregulated genes in MMAE‐resistant cells. Fig. S6. DPP4 mRNA levels in parental and MMAE‐resistant cells. Fig. S7. DPP4 mRNA levels in MMAE‐resistant cells after si‐DPP4 transfection. Fig. S8. Image of migration assay in MMAE‐resistant cells after si‐DPP4 transfection. Fig. S9. Image of invasion assay in MMAE‐resistant cells after si‐DPP4 transfection. Fig. S10. IC50 values of parental and MMAE‐resistant cells treated with sitagliptin. Fig. S11. Image of migration assay in parental and MMAE‐resistant cells after sitagliptin treatment. Fig. S12. Image of invasion assay in parental and MMAE‐resistant cells after sitagliptin treatment. Fig. S13. Cell proliferation according to XTT assay of parental cells after MMAE treatment. Fig. S14. Cell proliferation according to XTT assay of MMAE‐resistant cells after MMAE and sitagliptin treatment. Fig. S15. Western blotting of Bcl‐xL in parental cells after MMAE treatment. Fig. S16. Apoptosis assay in MMAE‐resistant cells after MMAE and/or sitagliptin treatment. Fig. S17. Western blotting of phospho‐AKT and AKT in parental cells after MMAE treatment. Fig. S18. Cell cycle assay in MMAE‐resistant cells after MMAE and/or sitagliptin treatment. Fig. S19. ROS assay of MMAE‐resistant cells after MMAE treatment. Fig. S20. Image of ROS assay in parental cells after MMAE treatment. Fig. S21. Image of ROS assay in MMAE‐resistant cells after MMAE and/or sitagliptin treatment. Fig. S22. Body weight changes in mice treated with MMAE and/or sitagliptin. [file MOL2-20-1347-s002.zip › Supplementary Figure.pdf]
