# Supplementary material for: Therapeutic strategies for MMAE‐resistant bladder cancer through DPP4 inhibition
Source: Mol Oncol. 2025 Dec 21;20(5):1347–63. doi: 10.1002/1878-0261.70187 (PMC13155154; doi:10.1002/1878-0261.70187)
Supplement: Supplementary file 1 — Fig. S1. Image of migration assay in parental and MMAE‐resistant cells after MMAE treatment. Fig. S2. Image of invasion assay in parental and MMAE‐resistant cells after MMAE treatment. Fig. S3. Apoptosis assay in parental and MMAE‐resistant cells after MMAE treatment. Fig. S4. Cell cycle assay in parental and MMAE‐resistant cells after MMAE treatment. Fig. S5. KEGG analysis of 702 upregulated genes in MMAE‐resistant cells. Fig. S6. DPP4 mRNA levels in parental and MMAE‐resistant cells. Fig. S7. DPP4 mRNA levels in MMAE‐resistant cells after si‐DPP4 transfection. Fig. S8. Image of migration assay in MMAE‐resistant cells after si‐DPP4 transfection. Fig. S9. Image of invasion assay in MMAE‐resistant cells after si‐DPP4 transfection. Fig. S10. IC50 values of parental and MMAE‐resistant cells treated with sitagliptin. Fig. S11. Image of migration assay in parental and MMAE‐resistant cells after sitagliptin treatment. Fig. S12. Image of invasion assay in parental and MMAE‐resistant cells after sitagliptin treatment. Fig. S13. Cell proliferation according to XTT assay of parental cells after MMAE treatment. Fig. S14. Cell proliferation according to XTT assay of MMAE‐resistant cells after MMAE and sitagliptin treatment. Fig. S15. Western blotting of Bcl‐xL in parental cells after MMAE treatment. Fig. S16. Apoptosis assay in MMAE‐resistant cells after MMAE and/or sitagliptin treatment. Fig. S17. Western blotting of phospho‐AKT and AKT in parental cells after MMAE treatment. Fig. S18. Cell cycle assay in MMAE‐resistant cells after MMAE and/or sitagliptin treatment. Fig. S19. ROS assay of MMAE‐resistant cells after MMAE treatment. Fig. S20. Image of ROS assay in parental cells after MMAE treatment. Fig. S21. Image of ROS assay in MMAE‐resistant cells after MMAE and/or sitagliptin treatment. Fig. S22. Body weight changes in mice treated with MMAE and/or sitagliptin. [file MOL2-20-1347-s002.zip › MO LG Supporting Information legend 251205.docx]

**Supporting Information legends**

Supplementary Figure 1

Representative image of migration assay in parental BC cells and MR-BCs treated with MMAE (T24/MR-T24: 20 nM; J82/MR-J82: 12 nM) (n = 3). Scale bar, 300 μm. MMAE, monomethyl auristatin E; MR-J82, MMAE-resistant J82; MR-T24, MMAE-resistant T24.

Supplementary Figure 2

Representative image of invasion assay in parental BC cells and MR-BCs treated with MMAE (T24/MR-T24: 20 nM; J82/MR-J82: 12 nM) (n = 8). Scale bar, 100 μm. MMAE, monomethyl auristatin E; MR-J82, MMAE-resistant J82; MR-T24, MMAE-resistant T24.

Supplementary Figure 3

Apoptosis assay using flow cytometry in parental BC cells and MR-BCs treated with MMAE (T24/MR-T24: 20 nM; J82/MR-J82: 12 nM) (n = 3). MMAE, monomethyl auristatin E; MR-J82, MMAE-resistant J82; MR-T24, MMAE-resistant T24.

Supplementary Figure 4

Cell cycle assay using flow cytometry in parental BC cells and MR-BCs treated with MMAE (T24/MR-T24: 20 nM; J82/MR-J82: 12 nM) (n = 3). The x-axis label “PE-A” represents the PI fluorescence signal. MMAE, monomethyl auristatin E; MR-J82, MMAE-resistant J82; MR-T24, MMAE-resistant T24.

Supplementary Figure 5-7

Supplementary Figure 5

KEGG analysis of 702 upregulated genes in MR-BCs. KEGG, Kyoto Encyclopedia of Genes and Genomes.

Supplementary Figure 6

*DPP4* mRNA levels in parental BC cells and MR-BCs, as determined by qRT-PCR (n = 4). The statistical test used was an unpaired *t*-test. *****p <* 0.0001. The error bars indicate standard deviation (SD). MR-J82, MMAE-resistant J82; MR-T24, MMAE-resistant T24.

Supplementary Figure 7

*DPP4* mRNA levels in MR-BCs after si-*DPP4* transfection, as determined by qRT-PCR (n = 4). The statistical test used was a one-way ANOVA. ns: no significance, ***p <* 0.01, *****p <* 0.0001. The error bars indicate SD. MR-J82, MMAE-resistant J82; MR-T24, MMAE-resistant T24.

Supplementary Figure 8

Representative image of migration assay in MR-BCs after transfection with si-*DPP4* (50 nM) (n = 3). Scale bar, 300 μm. MR-J82, MMAE-resistant J82; MR-T24, MMAE-resistant T24.

Supplementary Figure 9

Representative image of invasion assay in MR-BCs after transfection with si-*DPP4* (50 nM) (n = 8). Scale bar, 100 μm. MR-J82, MMAE-resistant J82; MR-T24, MMAE-resistant T24.

Supplementary Figure 10

IC_50_ values of parental BC cells and MR-BCs treated with sitagliptin (n = 4). The error bars indicate SD. MR-J82, MMAE-resistant J82; MR-T24, MMAE-resistant T24.

Supplementary Figure 11

Representative image of migration assay in parental BC cells and MR-BCs treated with sitagliptin (1.5 mM) (n = 3). Scale bar, 300 μm. MR-J82, MMAE-resistant J82; MR-T24, MMAE-resistant T24.

Supplementary Figure 12

Representative image of invasion assay in parental BC cells and MR-BCs treated with sitagliptin (1.5 mM) (n = 8). Scale bar, 100 μm. MR-J82, MMAE-resistant J82; MR-T24, MMAE-resistant T24.

Supplementary Figure 13-15

Supplementary Figure 13

Cell proliferation according to XTT assay after treatment of parental BC cells with MMAE (30 nM) (n = 8). The statistical test used was an unpaired *t*-test. ns: no significance, *****p <* 0.0001. The error bars indicate SD. MMAE, monomethyl auristatin E.

Supplementary Figure 14

Cell proliferation according to XTT assay after treatment of MR-BCs with MMAE and sitagliptin (n = 6). The error bars indicate SD. MMAE, monomethyl auristatin E; MR-J82, MMAE-resistant J82; MR-T24, MMAE-resistant T24.

Supplementary Figure 15

Protein levels of Bcl-xL in parental BC cells after treatment with MMAE (30 nM), as determined by western blotting (n = 3). MMAE, monomethyl auristatin E.

Supplementary Figure 16

Apoptosis assay using flow cytometry in MR-BCs treated with MMAE (30 nM) and/or sitagliptin (1.5 mM) (n = 3). MMAE, monomethyl auristatin E; MR-J82, MMAE-resistant J82; MR-T24, MMAE-resistant T24.

Supplementary Figure 17-22

Supplementary Figure 17

Protein levels of phospho-AKT and AKT in parental BC cells after treatment, as determined by western blotting (n = 3). MMAE, monomethyl auristatin E.

Supplementary Figure 18

Cell cycle assay using flow cytometry in MR-BCs treated with MMAE (30 nM) and/or sitagliptin (1.5 mM) (n = 3). The x-axis label “PE-A” represents the PI fluorescence signal. MMAE, monomethyl auristatin E; MR-J82, MMAE-resistant J82; MR-T24, MMAE-resistant T24.

Supplementary Figure 19

ROS assay of parental BC cells after MMAE (30 nM) and/or sitagliptin (1.5 mM) treatment (n = 3). The statistical test used was an unpaired *t*-test. ***p <* 0.01, *****p <* 0.0001. The error bars indicate SD. MMAE, monomethyl auristatin E.

Supplementary Figure 20

Representative image of ROS assay in parental BC cells treated with MMAE (30 nM) (n = 3). Scale bar, 300 μm. MMAE, monomethyl auristatin E.

Supplementary Figure 21

Representative image of ROS assay in MR-BCs treated with MMAE (30 nM) and/or sitagliptin (1.5 mM) (n = 3). Scale bar, 300 μm. MMAE, monomethyl auristatin E; MR-J82, MMAE-resistant J82; MR-T24, MMAE-resistant T24.

Supplementary Figure 22

Body weight changes in mice treated with MMAE (0.1 mg/kg) and/or sitagliptin (200 mg/kg) (n = 6). The statistical test used was a one-way ANOVA. ns: no significance. The error bars indicate SD. MMAE, monomethyl auristatin E.

Supplementary Table 1

Differentially expressed genes of MR-BCs versus parental BC cells.

Supplementary Table 2

qRT-PCR data of *DPP4* expression in parental BC cells and MR-BCs.

Supplementary Table 3

qRT-PCR data of *DPP4* expression in MR-BCs after si-*DPP4* transfection.
